# Quantitative mRNA expression measurement at home

**DOI:** 10.1038/s41598-023-49651-8

**Published:** 2024-01-10

**Authors:** Sonalisa Pandey, Sara Safa McCoy, Tsering Stobdan, Debashis Sahoo

**Affiliations:** 1 Shanvi, San Diego, CA USA; 2 Department of Pediatrics, University of California San Diego, 9500 Gilman Drive, MC 0703, Leichtag Building 132, La Jolla, CA 92093-0703 USA; 3 Department of Computer Science and Engineering, Jacob’s School of Engineering, University of California San Diego, La Jolla, USA

**Keywords:** Biological techniques, Molecular biology, Engineering

## Abstract

mRNA measurement is dominated by RT-PCR, which requires expensive laboratory equipment and personnel with advanced degrees. Loop-mediated isothermal amplification (LAMP) is a versatile technique for detecting target DNA and RNA. The sensitivity of LAMP in early reports has been below that of the standard RT-PCR tests. Here, we report the use of a fluorescence-based RT-LAMP protocol to measure CDX2 expression patterns, which match extremely well to the standards of sophisticated RT-PCR techniques (r = 0.99, p < 0.001). The assay works on diverse sample types such as cDNA, mRNA, and direct tissue sample testing in 25 min compared to more than 3 h for RT-PCR. We have developed a new protocol for designing RT-LAMP primers that reduce false positives due to self-amplification and improve quantification. A simple device with a 3D-printed box enables the measurement of mRNA expression at home, outdoors, and point-of-care setting.

## Introduction

Loop-mediated isothermal amplification (LAMP) protocols^1^ have become a prevalent method of developing diagnostic assays^2–14^. Diagnostics based on LAMP amplification are a compelling alternative to polymerase chain reaction (PCR) because LAMP can be performed without commercial thermocyclers, significantly reducing cost and time to result^1,15^. In addition, LAMP has advantages over PCR for targeting sequences due to its robustness against inhibitors^16,17^ and its high specificity, using four to six primers that identify six to eight regions on the template for amplification^1^. The simplicity of isothermal amplification enables translation to a simple point-of-care device based on disposable cartridges^9,10,18^. LAMP assay kits are commercially available, but almost all of them use LAMP for detection as opposed to reliable quantification directly from the sample^11,12,19–27^. There have been some attempts at reliable quantification using Reverse transcription- Loop-mediated isothermal amplification (RT-LAMP), which has advantages over Reverse transcription-polymerase chain reaction (RT-PCR), such as simpler instrumentation and faster reaction times^7,28,29^. However, it has gained less popularity for quantification as RT-PCR. One reason is that RT-PCR has been extensively validated and is widely accepted as a gold standard for quantification of RNA targets, which leads researchers and regulatory agencies to prefer RT-PCR for quantification^30,31^. Additionally, RT-LAMP is relatively new compared to RT-PCR; thus, there may be less familiarity with the technique and fewer established protocols. Besides, RT-LAMP may have some limitations in terms of specificity and sensitivity compared to RT-PCR, which could make quantification more challenging^11,32^.

Given all the above points, ongoing efforts are still to improve and standardize RT-LAMP for quantification purposes. As with any new technology, it may take time for RT-LAMP to gain wider acceptance and for researchers to become more comfortable with its use for quantification. Our goal is to evaluate if RT-LAMP can be used to measure gene expression values robustly and reliably and to address current challenges for RT-LAMP technologies, which include complex primer design steps and self-amplification of the primers that lead to false positive results. Here, we have developed a new protocol that improves previous primer design steps, and we estimate the time from fresh tissue harvesting to signal detection for gene expression quantification for two genes: caudal type homeobox transcription factor 2 (CDX2) and Actin beta (ACTB) using RT-LAMP. CDX2 is a master regulator of intestinal development and oncogenesis^33,34^; its expression is highly specific to the intestinal epithelium^35–37^ and is used as a diagnostic biomarker for colorectal cancer^38^. ACTB is a popular housekeeping genes that is highly expressed in almost all cells^39^. The methods proposed in this manuscript can be readily generalizable to many other genes.

## Results and discussion

### Overview of RT-LAMP assays and comparative experimental design with RT-PCR

**Figure 1:**
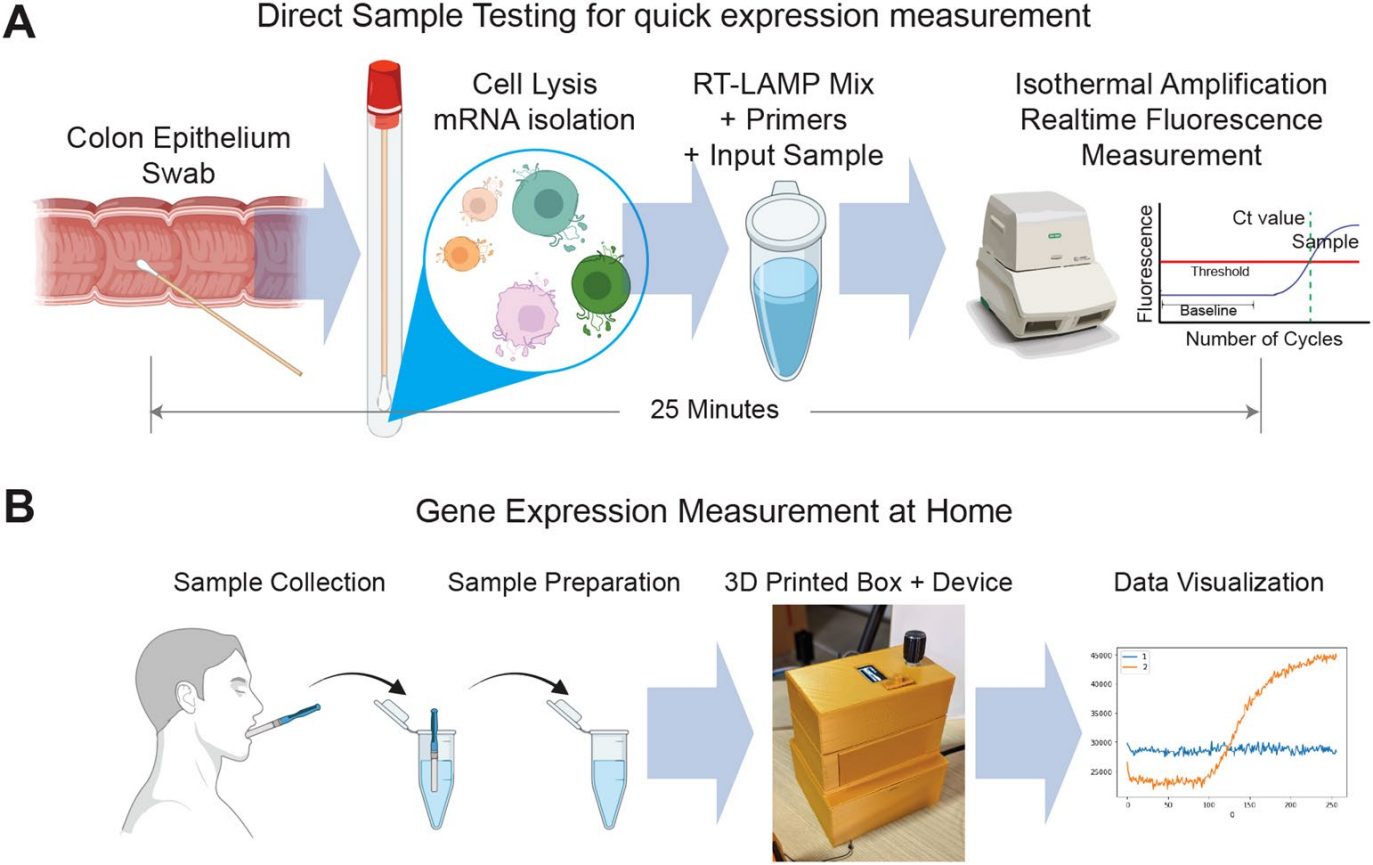
Workflow for faster measurement of mRNA expression. (**A**) Schematic displays of the proposed direct sample testing using RT-LAMP for quick-expression measurement in colon tissue. (**B**) Proposed device and workflow to demonstrate mRNA expression measurement at home.

We compared the RT-LAMP based approach directly with RT-PCR for measuring CDX2 and ACTB gene expression values (Fig. 1, Fig. S1). Schematic experimental designs to test RT-PCR and RT-LAMP data are presented in Fig. S1. We measured the time from sample collection to the fluorescent signal crossing the detection threshold for both genes in\ RT-PCR and RT-LAMP settings (Fig. 1A, Fig. S1). We also tested multiple tissue samples in human and mouse for tissue specific gene expression patterns (Fig. S1A). CDX2 is known to be colon tissue-specific, matched against RT-PCR and RT-LAMP data. Additionally, we tested diverse sample types, such as cDNA, mRNA, and Tissue QuickExtract, in both settings (Fig. S1B). We performed serial dilution experiment to check quantitative features for both RT-PCR and RT-LAMP data. We compared the Ct values using a correlation test (Fig. S1C). Our goal in this experiment was to evaluate whether RT-LAMP data can be used reliably to measure gene expression. We also tested if the RT-LAMP assay can be performed at home by building a 3D-printed device (Fig. 1B).

### CDX2 RT-LAMP expression is specific to colon tissue

**Figure 2:**
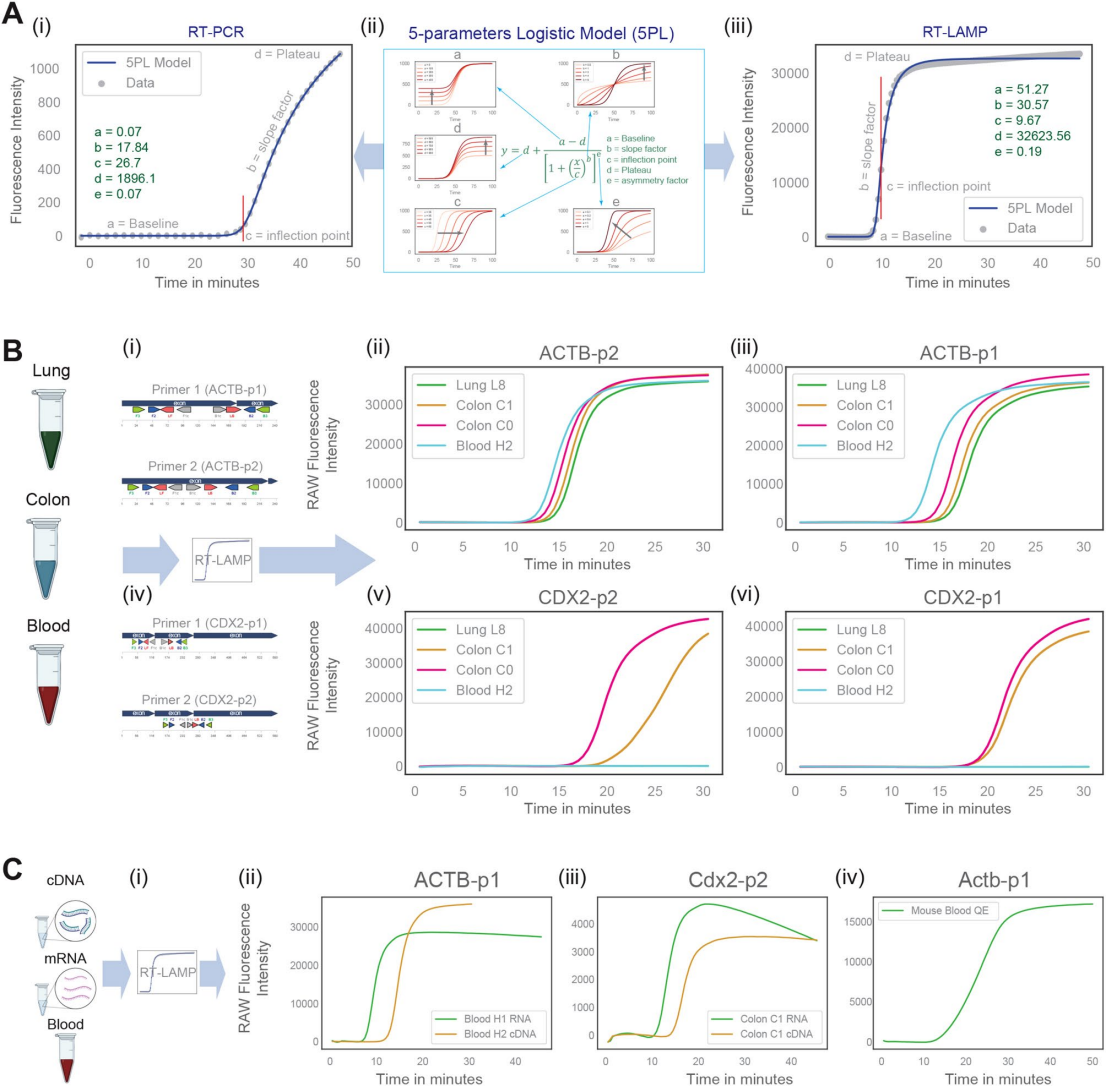
Rapid CDX2 expression measurement using RT-LAMP and comparison with RT-PCR. (**A**) Five parameter logistic (5PL) model for comparative analysis of RT-PCR and RT-LAMP data. 5PL model parameters: baseline (a), slope factor (b), inflection point (c), plateau (d), and asymmetry factor € are displayed for a sample RT-PCR (left) and RT-LAMP (right) data. The X-axis displays time in minutes, and the y-axis displays the raw fluorescence intensity. 5PL model is computed using least squares regression, and the model predicted data is shown as a blue line on top of the original raw data as grey dots. The inflection point is shown using a vertical red line. (**B**) RT-LAMP experiments using hACTB, ACTB2, CDX2, and hCDX2 LAMP primer sets on three different human tissues (lung, colon and blood) cDNA samples. (**C**) RT-LAMP experiment is performed on cDNA, mRNA, and Tissue quick extract samples. Welch's two sample two-tailed unpaired t-test is performed to compute the p values.

All four primers for the human gene ACTB and CDX2 were tested using RT-LAMP protocol (Fig. S2, Fig. 2) in four different human tissue cDNA samples (1 × Lung, 2 × Colon, 1 × Blood; Fig. 2C-i, iv) to check tissue-specific expression patterns. As expected, cDNA was amplified in both ACTB-p2 (Fig. 2B-ii) and ACTB-p1 (Fig. 2B-iii) primer sets. CDX2 cDNA was amplified only in the two colon samples used for CDX2-p2 (Fig. 2B-v) and CDX2-p1 primer sets (Fig. 2B-vi). Our data agree with previous findings that LAMP is highly specific^1,15,23^, and CDX2 is expressed specifically in the colon tissue^35–37^.

### RT-LAMP can be performed directly from cDNA, mRNA and tissue QuickExtract

To test the versatility of RT-LAMP, three different sample types were used: cDNA, mRNA, and tissue QuickExtract. RT-LAMP using ACTB-p2 primer set was able to amplify the target sequence using both mRNA and cDNA samples from human Blood (Fig. 2C-ii). Similarly, Cdx2-p2 primer set was able to amplify DNA using both mRNA and cDNA samples from mouse colon tissue (Fig. 2vC-iii). Next, we tested if gene expression can be measured directly from fresh tissue samples processed using Lucigen QuickExtract solution. Mouse blood was processed using Lucigen QuickExtract solution, and RT-LAMP protocol using Actb-p1 primer set was able to amplify Actb mRNA directly from the QuickExtract solution (Fig. 2C-iv). To validate this further, mouse colon, lung, kidney, spleen, and blood samples were used to test on all four mouse primer sets (Actb-p1, Actb-p2, Cdx2-p1, Cdx2-p2; Fig. S3A-i). As expected, Actb-p1 (Fig. S3A-ii) and Actb-p2 (Fig. S3A-iii) amplified all samples, while only the colon sample was amplified using Cdx2-p1 (Fig. S3A-iv) and Cdx2-p2 (Fig. S3A-v) primer set.

### RT-LAMP based gene expression matches well with RT-PCR

**Figure 3:**
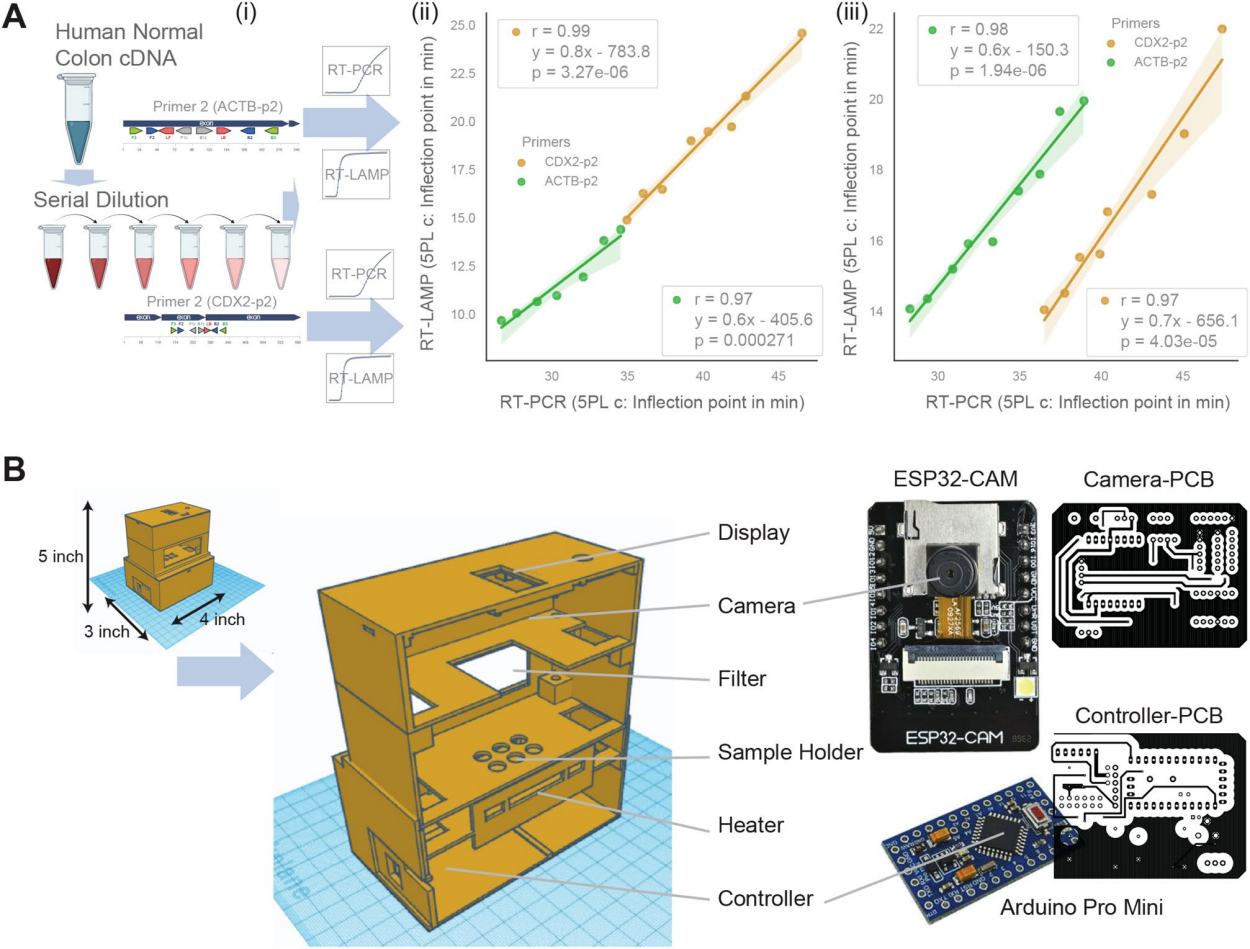
Correlation between RT-PCR and RT-LAMP. (**A**) Serial dilution was used to prepare human colon cDNA samples in various concentrations. hACTB and hCDX2 LAMP primer sets were used to perform RT-LAMP experiments from the diluted samples. F3 and B3 primers (hACTB and hCDX2) were used to perform RT-PCR experiments on the same diluted samples. The 5PL model was used to identify the inflection point for RT-LAMP and RT-PCR data and visualized using a scatter plot. This experiment was repeated in two human individuals’ cDNA samples (left and right). Correlation tests between RT-PCR and RT-LAMP data for both primers were calculated and displayed as scatter plots using Python seaborn lmplots with the p-values. The confidence interval around the regression line is indicated with shades. (**B**) 3D-model (4’’ × 3’’ × 5’’) of the 3D-printed box and device assembly details for use at home and outdoor settings. It uses an Arduino Pro Mini microcontroller and ESP32-CAM camera module to detect fluorescence signals. Results are displayed in a 0.96" 128X64 OLED LCD Display. Data from the device is collected over WIFI using an ESP32 module to a cell phone or a computer.

To check the accuracy of gene expression measurement using RT-LAMP, two independent serial dilutions of human cDNA samples were prepared and processed using both RT-LAMP and RT-PCR protocols (Fig. 3A-i). For RT-LAMP protocol CDX2-p2 and ACTB-p2 primer sets were used, while RT-PCR protocol used only the F3 and B3 primers from the CDX2-p2 and ACTB-p2 primer sets. Both RT-PCR and RT-LAMP data were modeled using the five-parameters logistic (5PL) function, and all parameters were estimated using logistic regression^40,41^. The inflection points for RT-PCR and RT-LAMP were highly correlated for both CDX2-p2 (r = 0.99, p = 1.27e-05, Fig. 3A-ii) and ACTB-p2 (r = 0.97, p = 0.000271, Fig. 3A-ii). Another independent serial dilution experiment showed similar results for both CDX2-p2 (r = 0.97, p = 4.03e-05, Fig. 3A-iii) and ACTB-p2 (r = 0.98, p = 1.94e −05, Fig. 3A-iii). The results in cDNA quantification need to be repeated using mRNA quantification to check the overall performance of the RT-LAMP assay compared to RT-PCR in the future. Our results suggest that CDX2 expression can be reliably measured using RT-LAMP. While in our assay, RT-LAMP was faster than RT-PCR (Fig. S4), it may not be generalizable as the optimization in both settings may lead to variable results. The quantification results may be generalizable to fundamental nucleic acid detection using RT-LAMP techniques.

### mRNA expression can be performed at home

To demonstrate if mRNA expression measurement can be performed outside a laboratory setting, we build a simple and affordable device (length = 4 in, width = 3 in, height = 5 in) that can be built using commercially available components (Fig. 3B). The camera can be easily replaced with a cell phone camera which can collect, analyze, and share data efficiently. CDX2-p2 RT-LAMP primers successfully quantified mRNA data at home (Fig. S5A), in laboratory benches (Fig. S5B), and in outdoor settings (Fig. S5C). This work demonstrates that mRNA expression measurement will not be limited to laboratory settings only. Consumer access to this assay and data, enabling innovative healthcare and hygiene applications. This technology will significantly reduce the expertise of the personnel needed to operate the device and quantify mRNA expression.

### Modified RT-LAMP primer design steps to improve quantification and reduce false positive

One of the major challenges for the RT-LAMP is the complex primer design steps, which frequently produce primers that can self-amplify given enough time without any input sample^42^. Self-amplification will lead to false positives in the result. Efficient quantification of RT-LAMP technologies depends on quick amplification of input nucleic acid and delayed or no self-amplification. We found a new relationship between efficient quantification and regular PCR amplification using F3/B3 part of the RT-LAMP primer (Fig. S6A). The quantification of RT-LAMP is jointly determined by primer sets, and the influence of inner primers should also be considered. Our data suggest that the stronger the PCR using the F3/B3, the better the quantification of RT-LAMP (CDX2-p2, Ct = 25, Correlation coefficient = 0.99) in a serial dilution experiment. CDX2-p2 performed exceptionally well in all experiments compared to other primers. Amplification of DNA is visualized by GEL electrophoresis for both RT-PCR (Fig. S6B) and RT-LAMP (Fig. S6C) in control, colon cDNA sample and serial dilution experiment. Detailed analysis of a self-amplifying mouse Actb DNA-only LAMP primer set revealed that both FIP and BIP were the culprit (Fig. S6D). BIP self-amplified slowly, FIP self-amplified sharply after 25 min, whereas F3 + B3 did not self-amplify. A software algorithm to filter the FIP and BIP sequences can be carried out by searching for hairpin structures that enables self-amplification. RT-LAMP primers can be screened alongside the standard primer design process for stronger F3/B3 PCR and weak/no self-amplification to identify the best reagents for mRNA quantification. This strategy will make it convenient to identify a highly quantitative primer set. In the future, this process can be automated by developing appropriate machine learning models.

## Conclusion

In summary, this work leveraged the advantages of the Loop Mediated Isothermal Amplification (LAMP) to develop a molecular diagnostic assay based on CDX2 expression. Cotton swab-based sample collection and direct sample testing procedures were developed, which were demonstrated to be superior to whole tissue chunks. The time from sample collection to CDX2 expression data was about 25 min. Optimization on the primer set could potentially reduce this time further. A 10–25-min diagnostic assay at the point of care setting will improve health care broadly across many diseases. This will also enable the development of consumer devices for at-home molecular testing. We hope this result will galvanize the community to develop innovative molecular testing both at home and at point of care settings.

### Supplementary Information

Supplementary Information 1.

Supplementary Information 2.

Supplementary Information 3.

Supplementary Information 4.

## Data Availability

All data and materials are available in the main text or the supplementary materials. All codes used in the analysis are available in the github:sahoo00/LAMP.
