# Supplementary material for: Quantitative mRNA expression measurement at home
Source: Sci Rep. 2024 Jan 10;14:1013. doi: 10.1038/s41598-023-49651-8 (PMC10781964; doi:10.1038/s41598-023-49651-8)

**Supplementary Data 1: Primer Design (8 pages)**

CDX2 Primer 1 – CDX2

| **Name** | **Sequence** | **Scale** | **Purification** |
| --- | --- | --- | --- |
| CDX2-F3 | CGCCGAGCAGCTGTCT | 25nmS | STD |
| CDX2-B3 | GCTAGCTCGGCTTTCCTC | 25nmS | STD |
| CDX2-FIP | TTGGCTGCCGAGGGACTGCCAGCGGCGGAACCTGT | 25nmS | STD |
| CDX2-BIP | TCGAGTGGTGTACACGGACCACGGATGGTGATGTAGCGACTG | 25nmS | STD |
| CDX2-LF | GGCTTCCGCATCCACTC | 25nmS | STD |
| CDX2-LB | CGGCTGGAGCTGGAGAA | 25nmS | STD |
| F2 | CAGCGGCGGAACCTGT |  |  |
| F1c | TTGGCTGCCGAGGGACTGC |  |  |
| B2 | GGATGGTGATGTAGCGACTG |  |  |
| B1c | TCGAGTGGTGTACACGGACCAC |  |  |


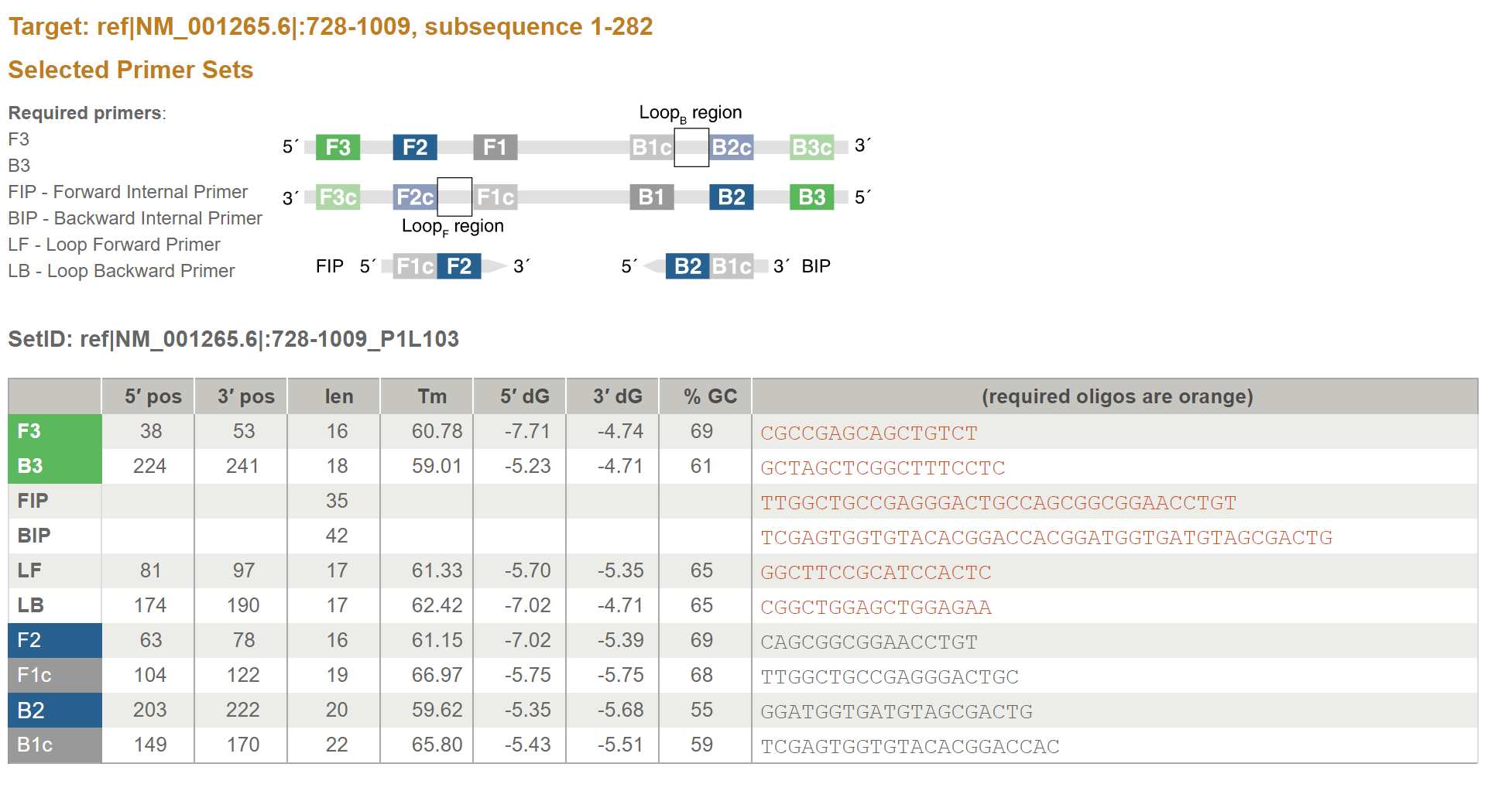


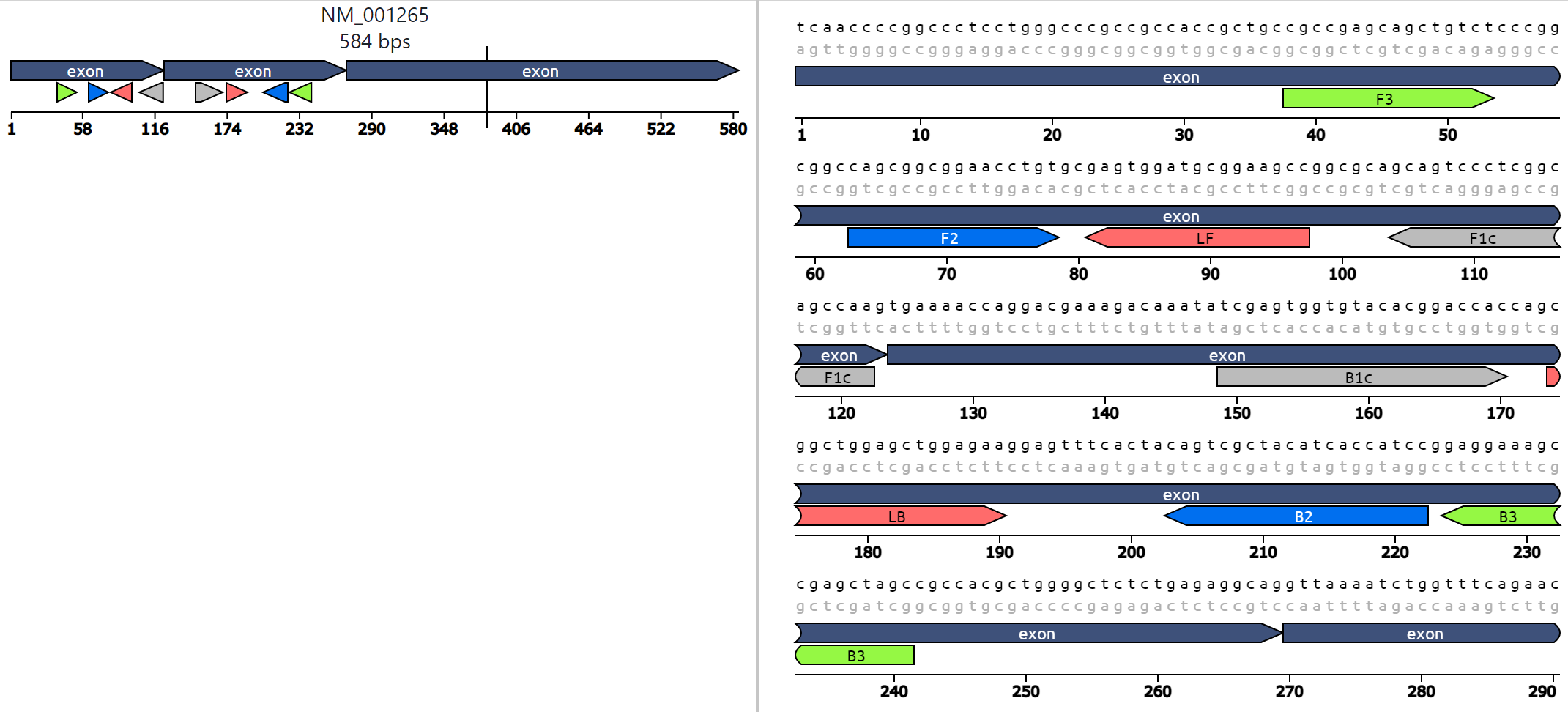


CDX2 Primer 2 - hCDX2

| **Name** | **Sequence** | **Scale** | **Purification** |
| --- | --- | --- | --- |
| hCDX2F3 | GGTGTACACGGACCACCA | 25nmS | STD |
| hCDX2B3 | GCTGCTGCAACTTCTTCTTG | 25nmS | STD |
| hCDX2FIP | CTCGGCTTTCCTCCGGATGGCTGGAGCTGGAGAAGGAGTT | 25nmS | STD |
| hCDX2BIP | ACGCTGGGGCTCTCTGAGACTCTCCTTTGCTCTGCGGTT | 25nmS | STD |
| hCDX2LB | GGCAGGTTAAAATCTGGTTTCA | 25nmS | STD |
| F2 | CTGGAGCTGGAGAAGGAGTT |  |  |
| F1c | CTCGGCTTTCCTCCGGATGG |  |  |
| B2 | CTCTCCTTTGCTCTGCGGTT |  |  |
| B1c | ACGCTGGGGCTCTCTGAGA |  |  |


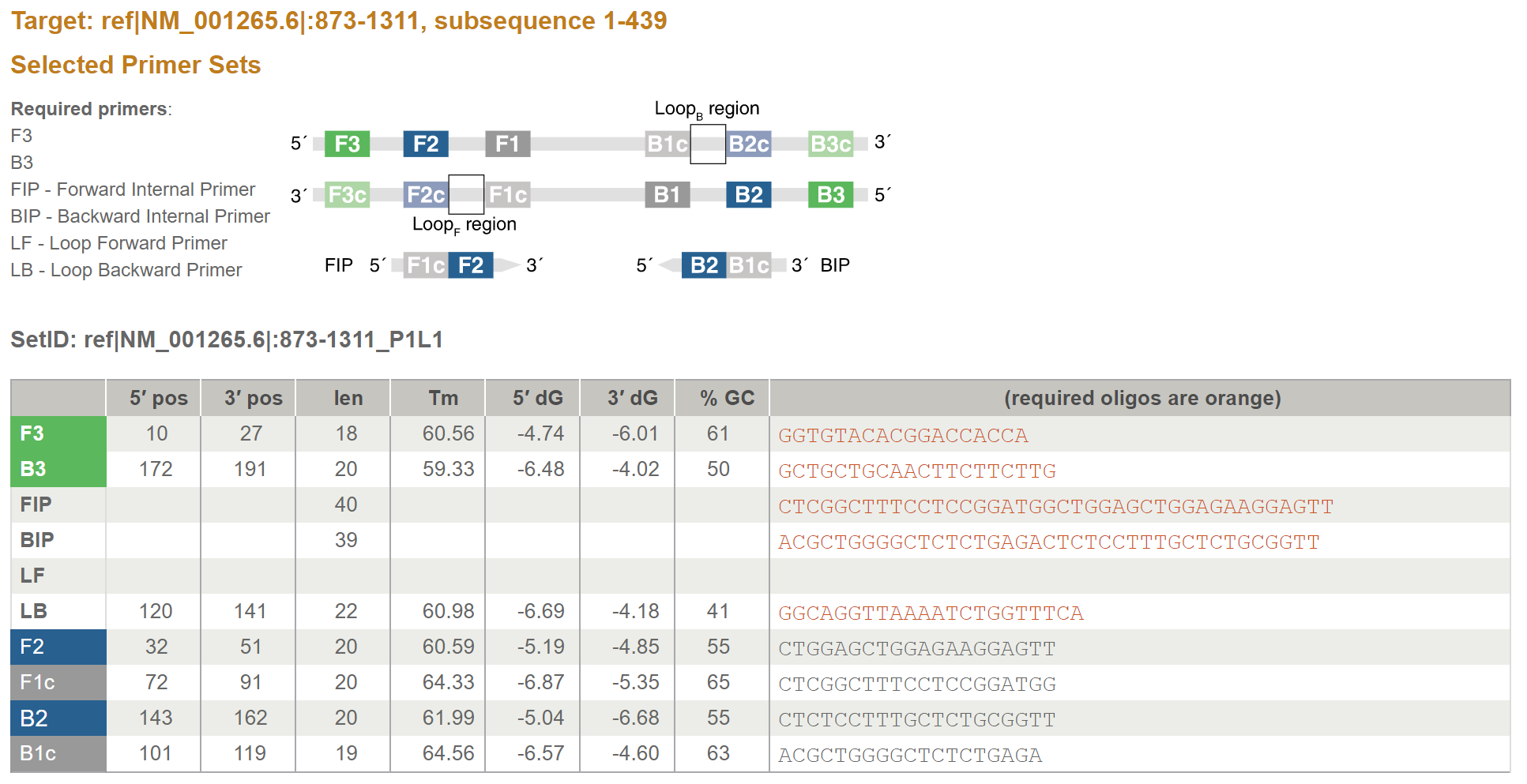


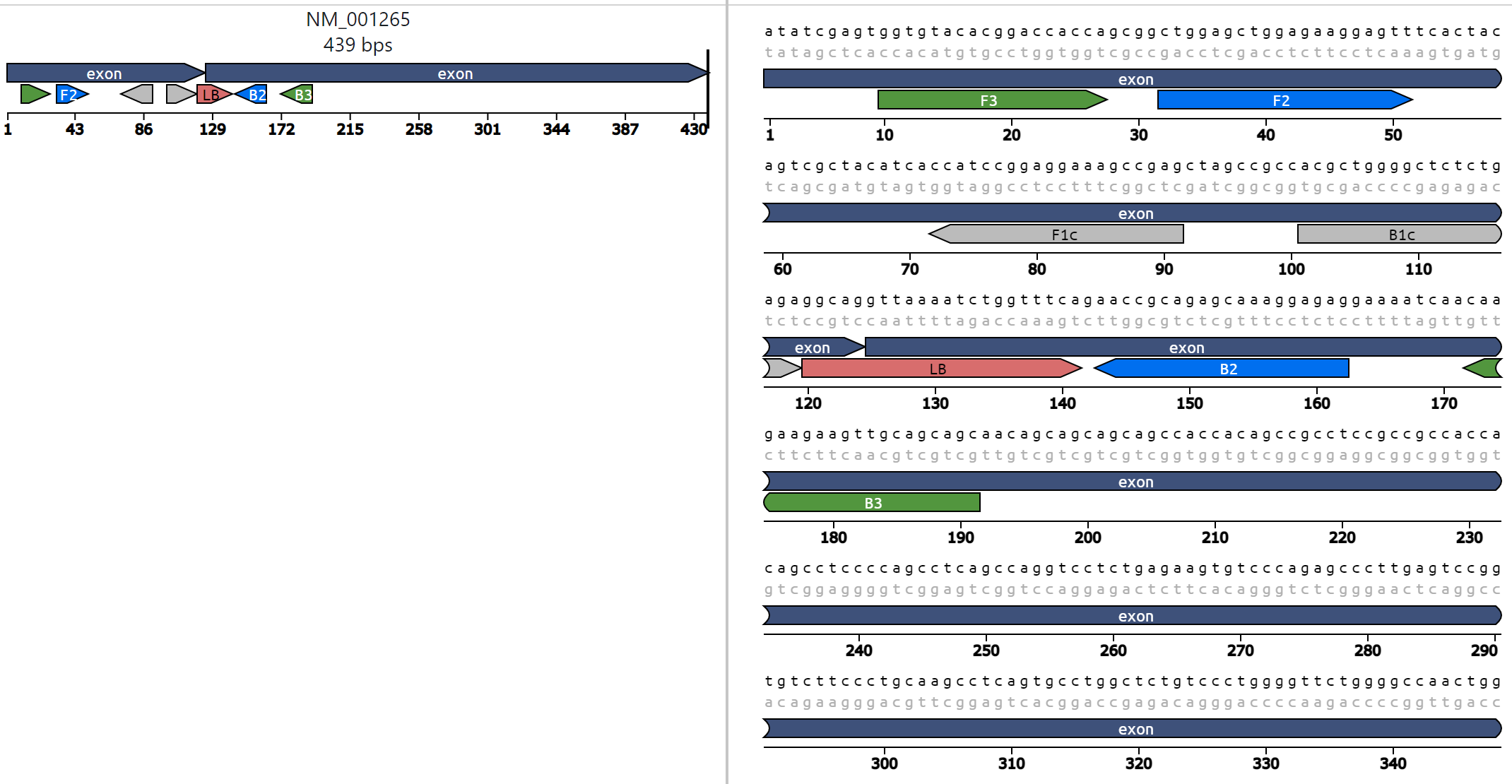


ACTB Primer 1 (ACTB2)

| **Name** | **Sequence** | **Scale** | **Purification** |
| --- | --- | --- | --- |
| ACTB2-F3 | AGTACCCCATCGAGCACG | 25nmS | STD |
| ACTB2-B3 | AGCCTGGATAGCAACGTACA | 25nmS | STD |
| ACTB2-FIP | GAGCCACACGCAGCTCATTGTATCACCAACTGGGACGACA | 25nmS | STD |
| ACTB2-BIP | CTGAACCCCAAGGCCAACCGGCTGGGGTGTTGAAGGTC | 25nmS | STD |
| ACTB2-LF | TGTGGTGCCAGATTTTCTCCA | 25nmS | STD |
| ACTB2-LB | CGAGAAGATGACCCAGATCATGT | 25nmS | STD |
| F2 | TCACCAACTGGGACGACA |  |  |
| F1c | GAGCCACACGCAGCTCATTGTA |  |  |
| B2 | GCTGGGGTGTTGAAGGTC |  |  |
| B1c | CTGAACCCCAAGGCCAACCG |  |  |


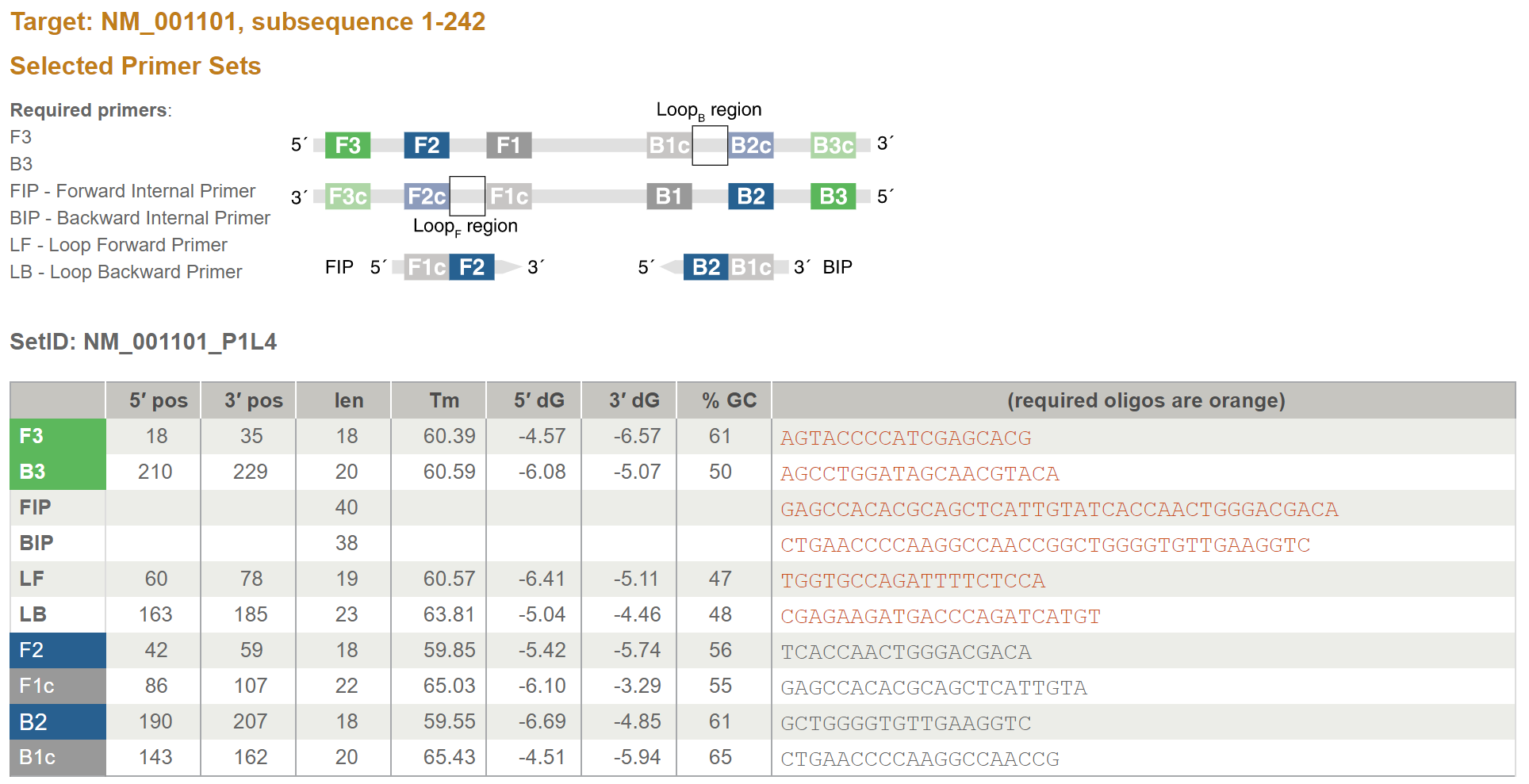


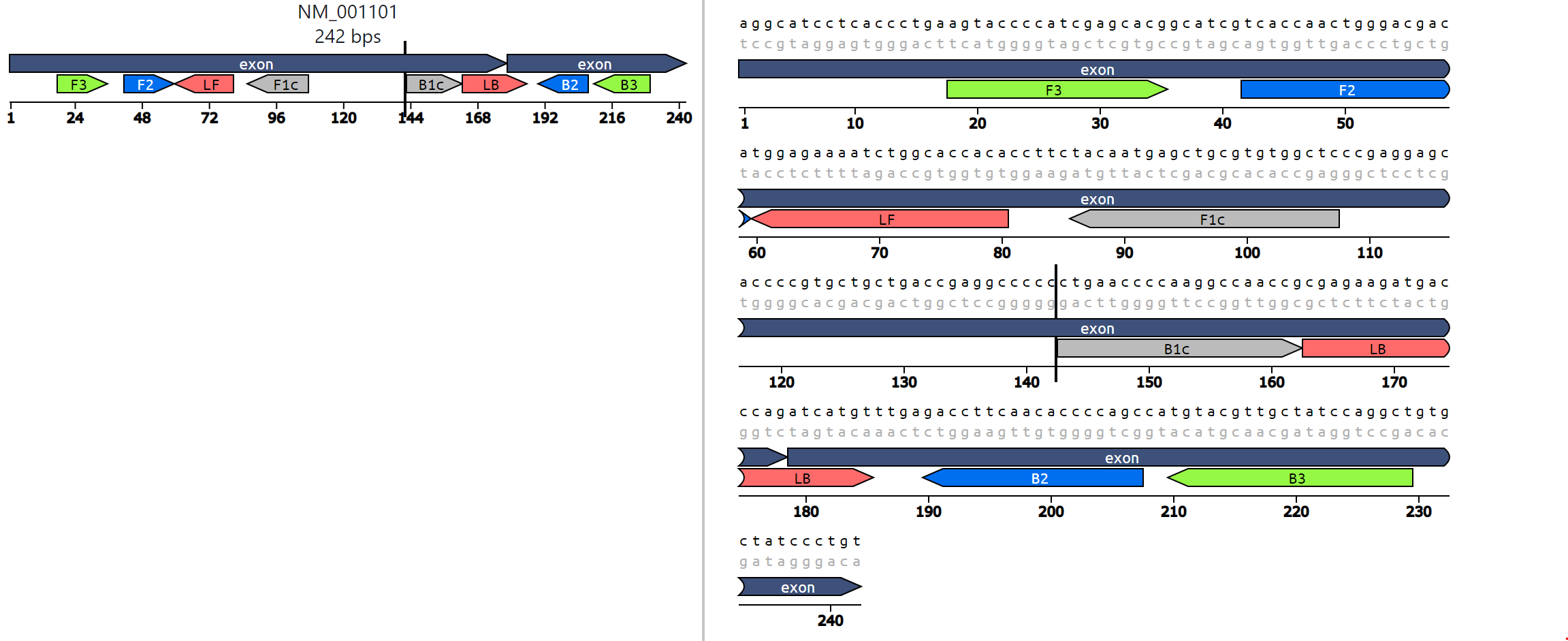


ACTB Primer 2 (hACTB)

| **Name** | **Sequence** | **Scale** | **Purification** |
| --- | --- | --- | --- |
| hACTB-F3 | GCGCGGCTACAGCTTCA | 25nmS | STD |
| hACTB-B3 | GGAAGAGTGCCTCAGGGC | 25nmS | STD |
| hACTB-FIP | AAGTCCAGGGCGACGTAGCACCGGCCGAGCGGGAAAT | 25nmS | STD |
| hACTB-BIP | GAGATGGCCACGGCTGCTTCCATTGCCAATGGTGATGACCT | 25nmS | STD |
| hACTB-LF | TTCTCCTTAATGTCACGCACG | 25nmS | STD |
| hACTB-LB | CCCTGGAGAAGAGCTACGAG | 25nmS | STD |
| F2 | CGGCCGAGCGGGAAAT |  |  |
| F1c | AAGTCCAGGGCGACGTAGCAC |  |  |
| B2 | TTGCCAATGGTGATGACCT |  |  |
| B1c | GAGATGGCCACGGCTGCTTCCA |  |  |


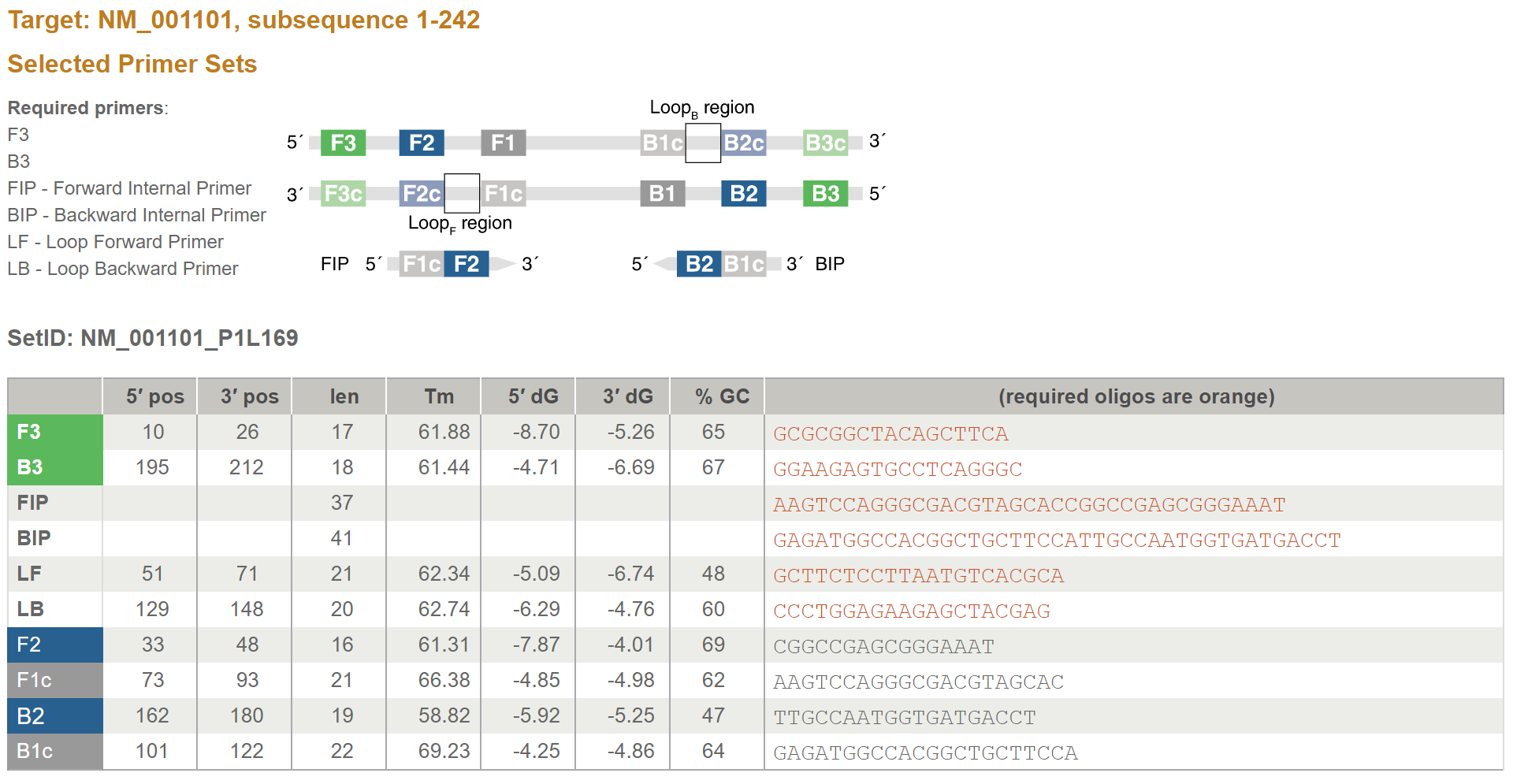


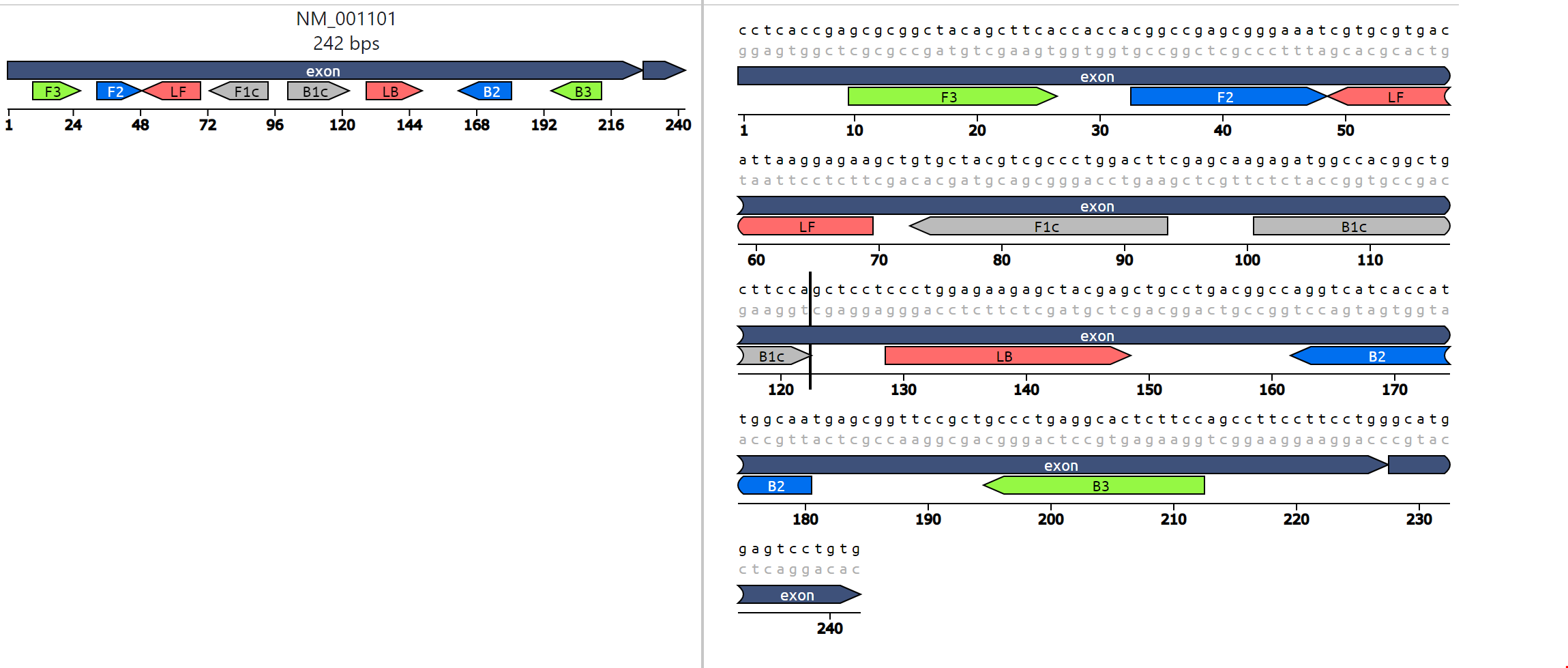


Mouse Actb Primer 1 (Actb)

ref|NM_007393.5|:983-1238, subsequence 1-256

F3 34 51 18 60.83 -6.00 -6.69 61 ACCACCATGTACCCAGGC

B3 209 226 18 60.08 -7.97 -4.90 61 GGCCGGACTCATCGTACT

FIP 42 GCGCTCAGGAGGAGCAATGATCATTGCTGACAGGATGCAGAA

BIP 42 TACTCTGTGTGGATCGGTGGCTTGCTTGCTGATCCACATCTG

LF 83 100 18 61.92 -6.41 -5.19 61 TGGTGCTAGGAGCCAGAG

LB 167 185 19 62.56 -5.35 -4.86 58 CCTCACTGTCCACCTTCCA

F2 52 71 20 59.29 -4.79 -5.26 45 ATTGCTGACAGGATGCAGAA

F1c 111 132 22 65.21 -7.27 -3.80 59 GCGCTCAGGAGGAGCAATGATC

B2 187 206 20 59.90 -5.41 -4.40 50 TGCTTGCTGATCCACATCTG

B1c 136 157 22 65.07 -3.82 -6.24 55 TACTCTGTGTGGATCGGTGGCT


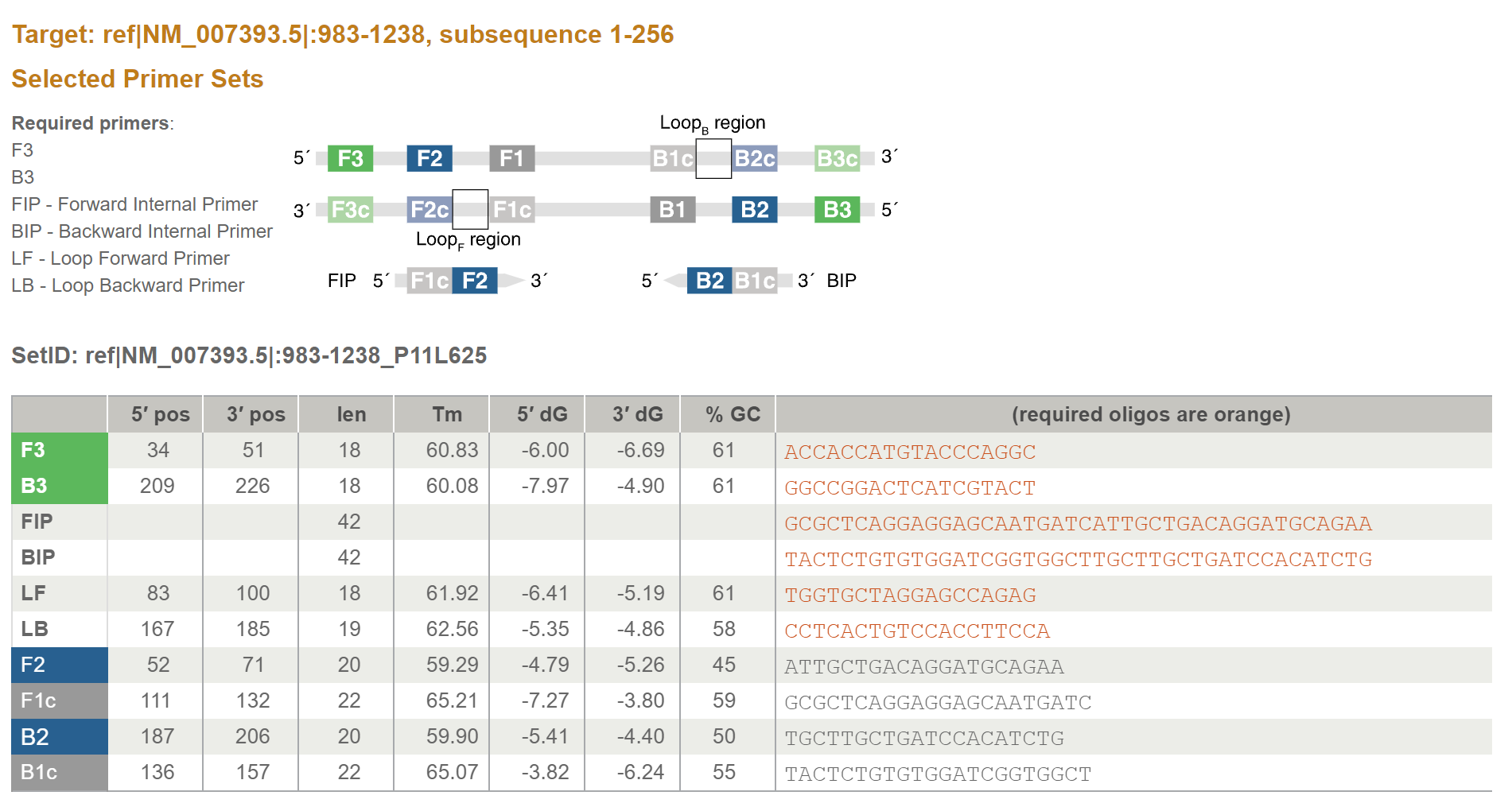


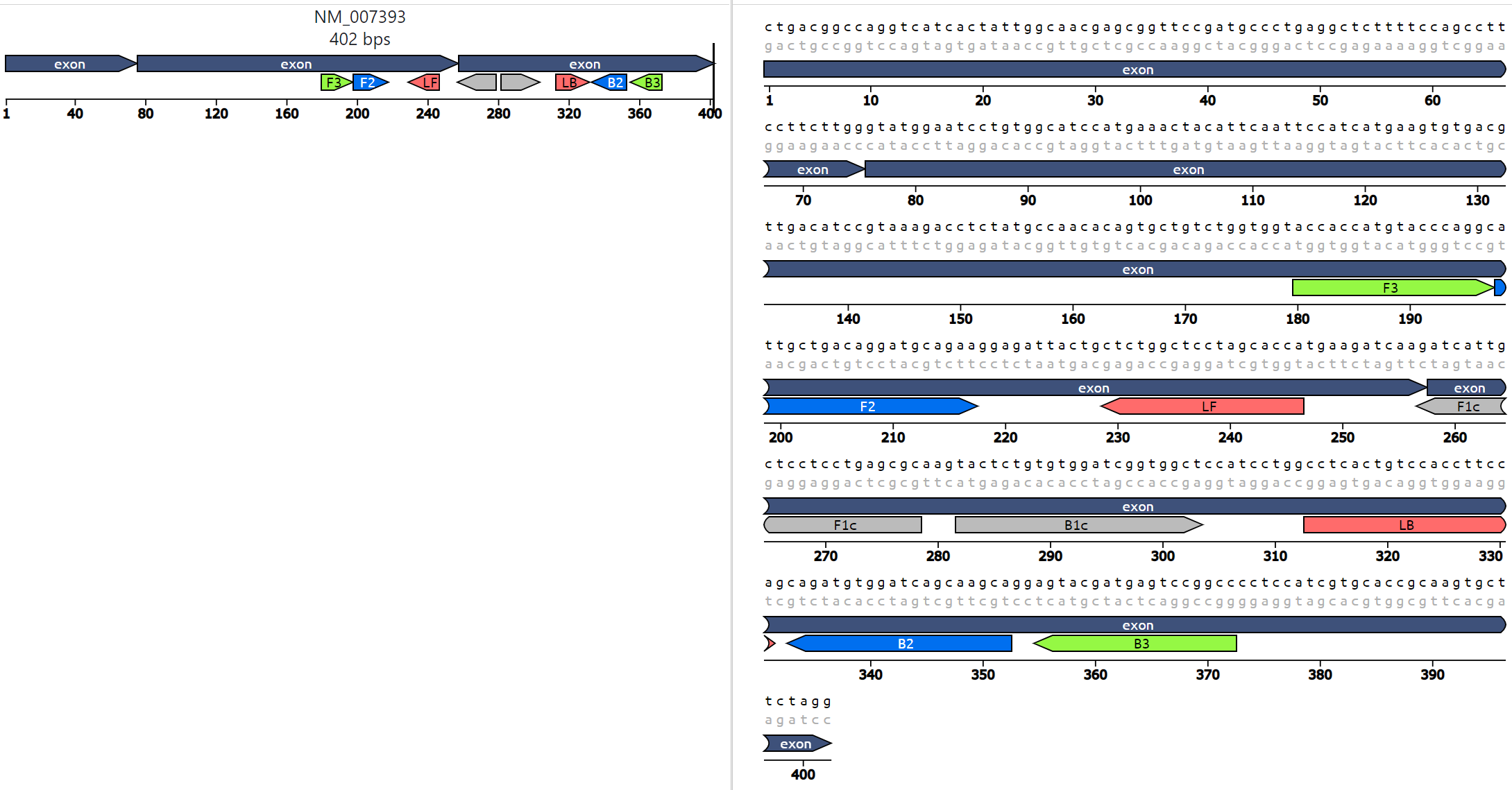


Mouse Actb Primer 2 (Actb2)

SetID: ref|NM_007393.5|:837-1181_P1L1

5′ pos 3′ pos len Tm 5′ dG 3′ dG % GC (required oligos are orange)

F3 85 104 20 59.93 -5.50 -4.01 50 CCTGTGGCATCCATGAAACT

B3 306 323 18 60.17 -5.35 -4.74 61 GGACAGTGAGGCCAGGAT

FIP 42 CTGGGTACATGGTGGTACCACCAAGTGTGACGTTGACATCCG

BIP 41 TACTGCTCTGGCTCCTAGCACCAGAGTACTTGCGCTCAGGA

LF 143 164 22 61.07 -4.72 -3.10 41 TGTGTTGGCATAGAGGTCTTTA

LB 247 268 22 60.02 -4.27 -6.10 41 TGAAGATCAAGATCATTGCTCC

F2 123 142 20 59.94 -4.55 -5.68 50 AAGTGTGACGTTGACATCCG

F1c 174 195 22 64.20 -5.84 -6.00 59 CTGGGTACATGGTGGTACCACC

B2 269 287 19 59.77 -3.82 -5.11 53 AGAGTACTTGCGCTCAGGA

B1c 224 245 22 65.40 -4.98 -6.24 59 TACTGCTCTGGCTCCTAGCACC


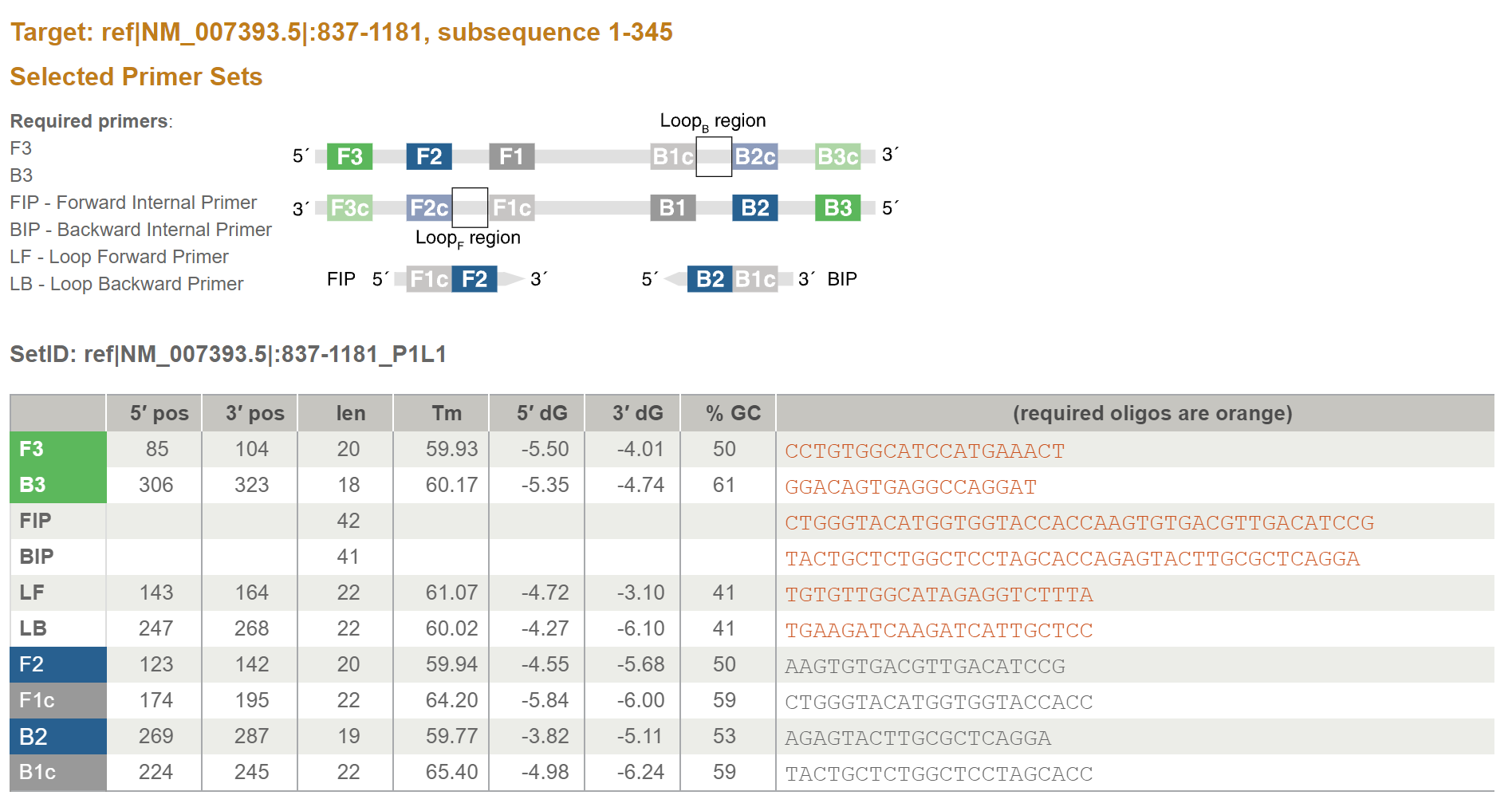


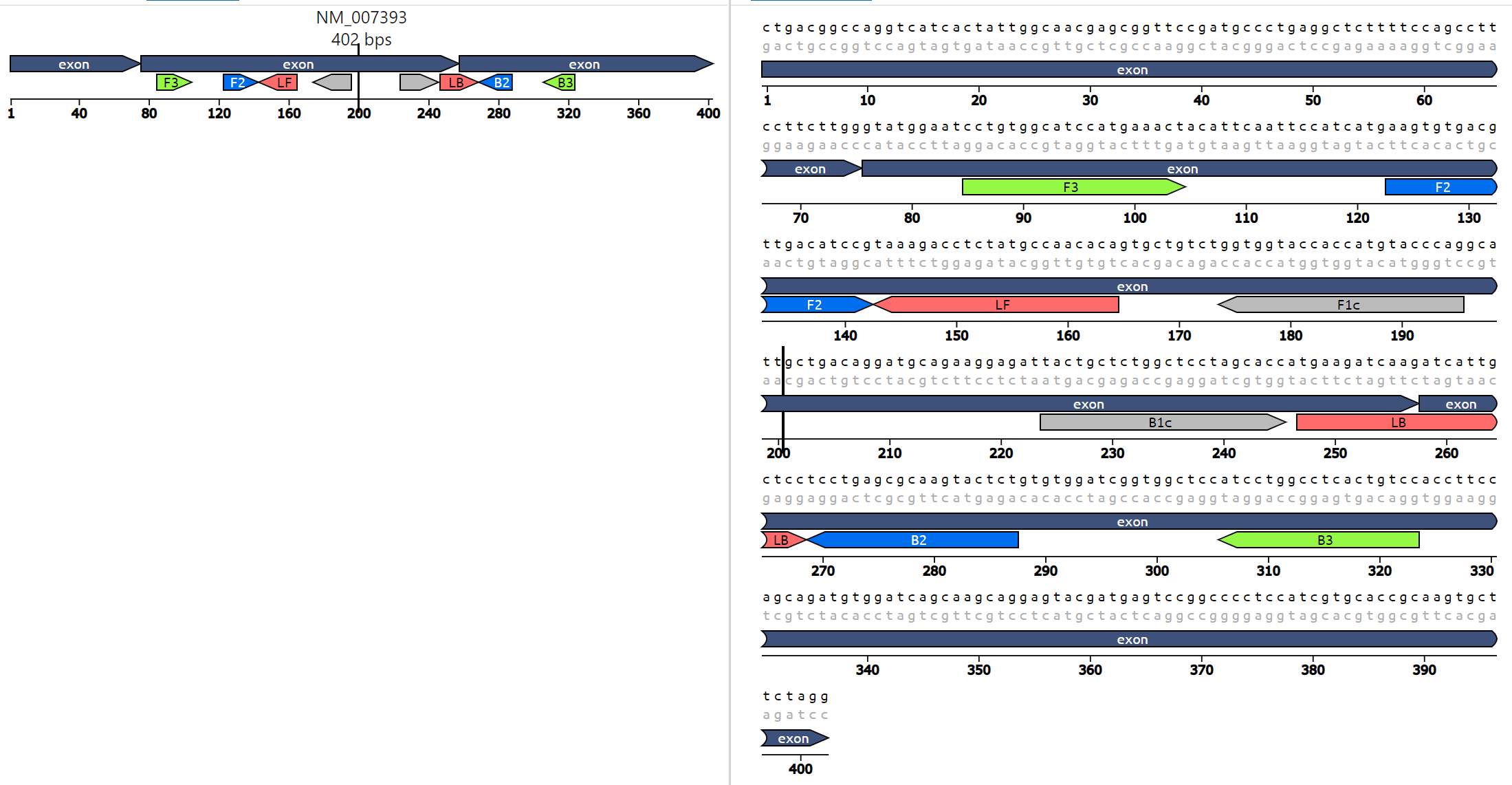


Mouse Cdx2 Primer 1 (Cdx2)

SetID: ref|NM_007673.3|:748-1059_P1L6

5′ pos 3′ pos len Tm 5′ dG 3′ dG % GC (required oligos are orange)

F3 6 24 19 59.65 -4.50 -4.91 53 AAACCTGTGCGAGTGGATG

B3 180 197 18 60.63 -5.88 -5.05 61 TCGGAGAGCCCAAGTGTG

FIP 40 GGTCTGTGTACACCACCCGGTAGCAGTCCCTAGGAAGCCA

BIP 40 GGCTGGAGCTGGAGAAGGAGTGCCAGCTCACTTTTCCTCC

LF 58 79 22 61.06 -4.41 -4.18 41 TGTCTTTTGTCCTGGTTTTCAC

LB 133 157 25 62.49 -4.16 -4.91 40 CACTTTAGTCGATACATCACCATCA

F2 39 56 18 60.14 -5.75 -5.75 61 GCAGTCCCTAGGAAGCCA

F1c 82 103 22 65.50 -5.35 -5.86 59 GGTCTGTGTACACCACCCGGTA

B2 158 176 19 60.04 -7.09 -5.55 58 GCCAGCTCACTTTTCCTCC

B1c 110 130 21 65.59 -6.69 -5.08 62 GGCTGGAGCTGGAGAAGGAGT


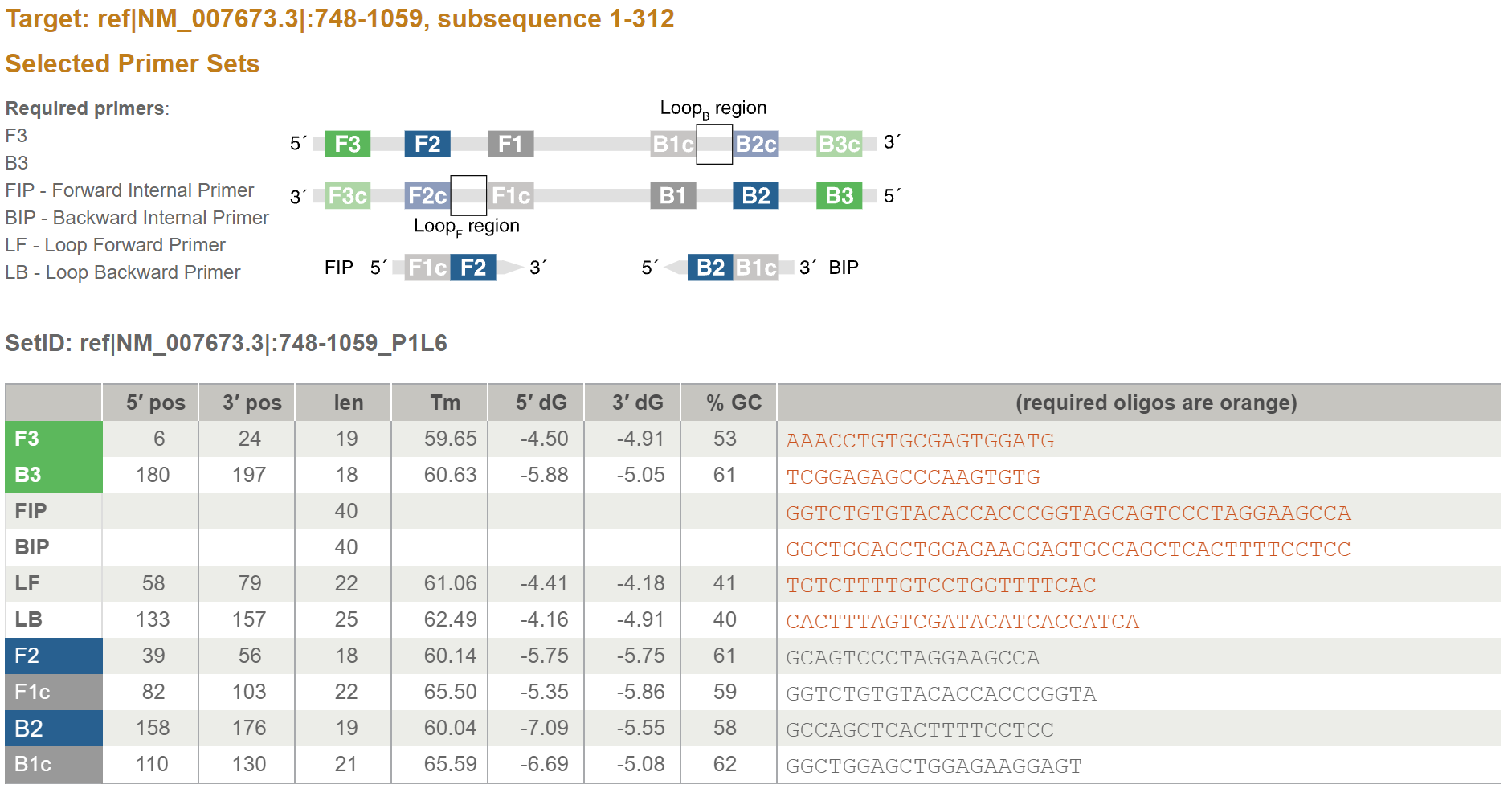


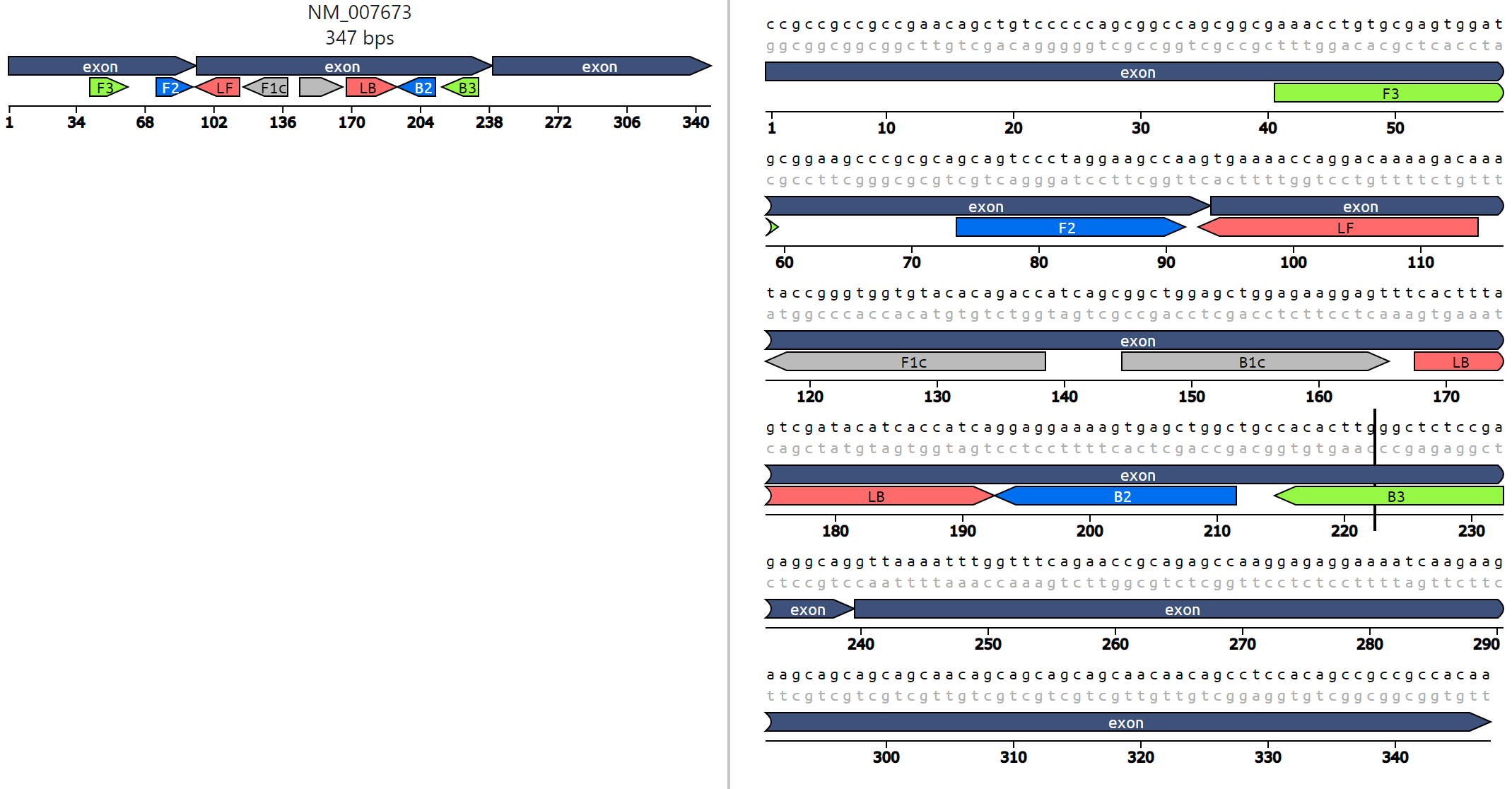


Mouse Cdx2 Primer 2 (mCdx2)

SetID: ref|NM_007673.3|:713-1053_P1L6

5′ pos 3′ pos len Tm 5′ dG 3′ dG % GC (required oligos are orange)

F3 34 54 21 69.16 -7.76 -6.42 62 AGCGGCGAAACCTGTGCGAGT

B3 215 232 18 60.63 -5.88 -5.05 61 TCGGAGAGCCCAAGTGTG

FIP 40 GGTCTGTGTACACCACCCGGTAGCAGTCCCTAGGAAGCCA

BIP 40 GGCTGGAGCTGGAGAAGGAGTGCCAGCTCACTTTTCCTCC

LF 93 114 22 61.06 -4.41 -4.18 41 TGTCTTTTGTCCTGGTTTTCAC

LB 168 192 25 62.49 -4.16 -4.91 40 CACTTTAGTCGATACATCACCATCA

F2 74 91 18 60.14 -5.75 -5.75 61 GCAGTCCCTAGGAAGCCA

F1c 117 138 22 65.50 -5.35 -5.86 59 GGTCTGTGTACACCACCCGGTA

B2 193 211 19 60.04 -7.09 -5.55 58 GCCAGCTCACTTTTCCTCC

B1c 145 165 21 65.59 -6.69 -5.08 62 GGCTGGAGCTGGAGAAGGAGT


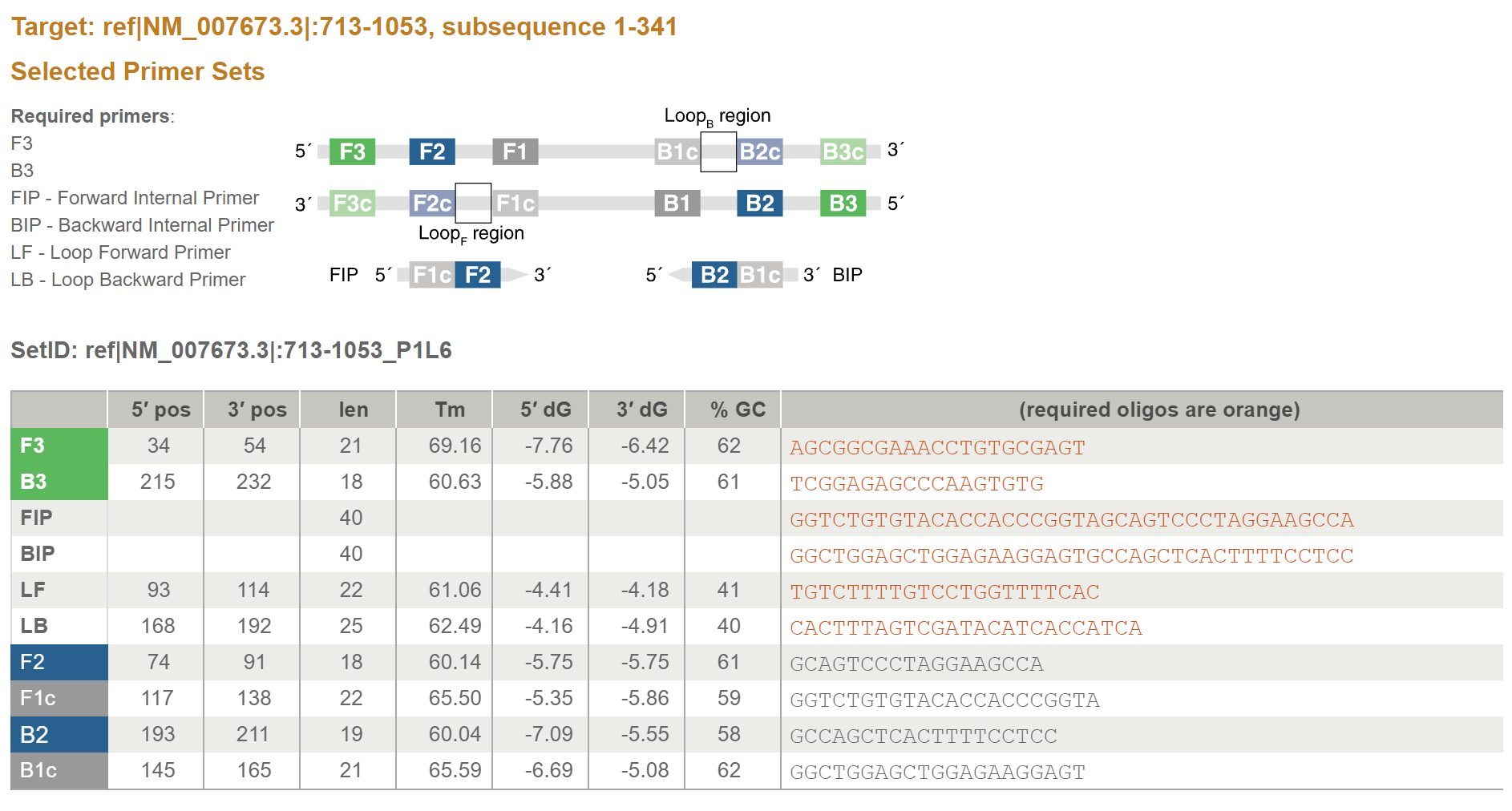


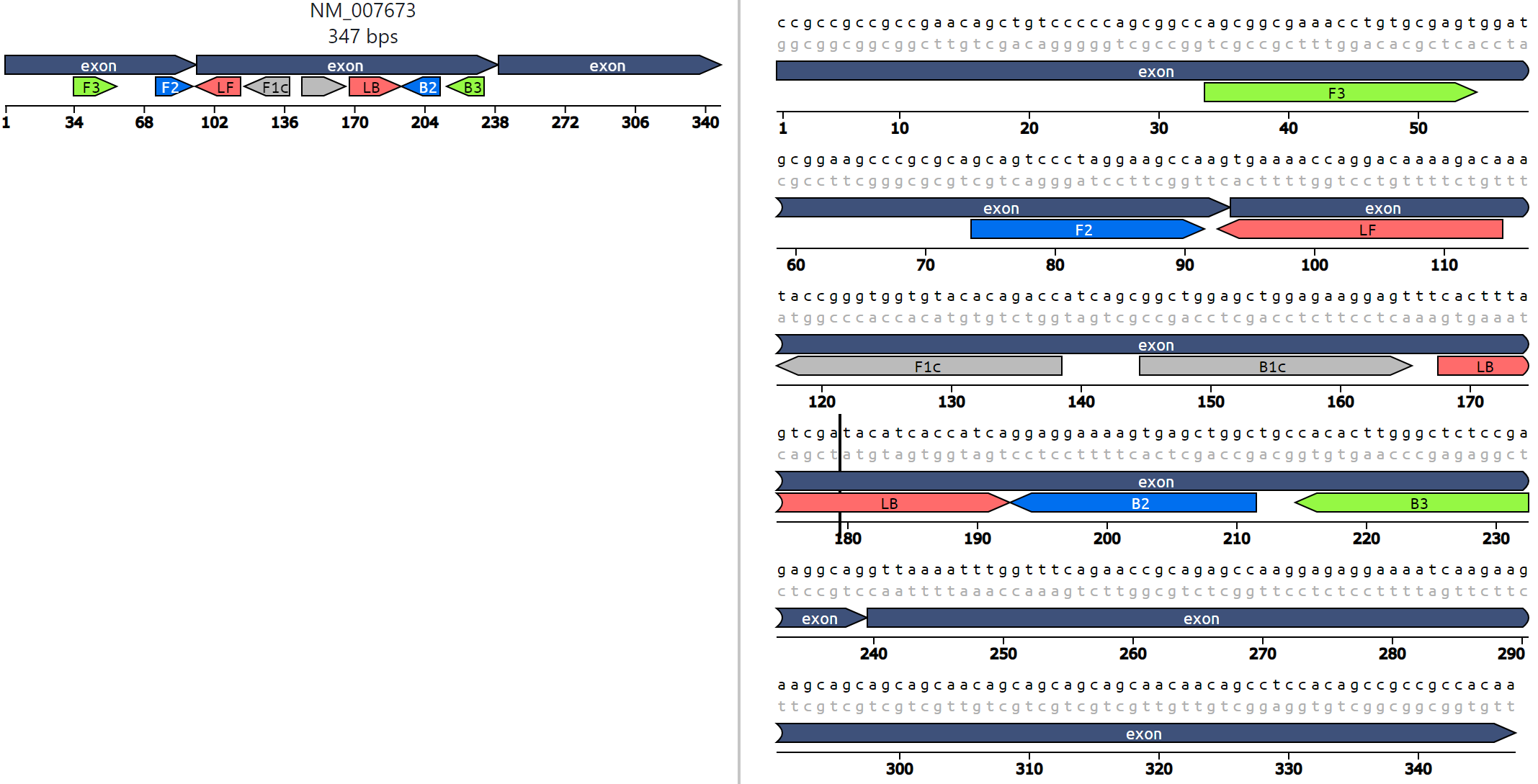

Supplement: Supplementary file 1 — Supplementary Information 1. [file 41598_2023_49651_MOESM1_ESM.docx]
