# Supplementary figures and images for: Quantitative mRNA expression measurement at home
Source: Sci Rep. 2024 Jan 10;14:1013. doi: 10.1038/s41598-023-49651-8 (PMC10781964; doi:10.1038/s41598-023-49651-8)

Supplementary Data 2: Circuit Design

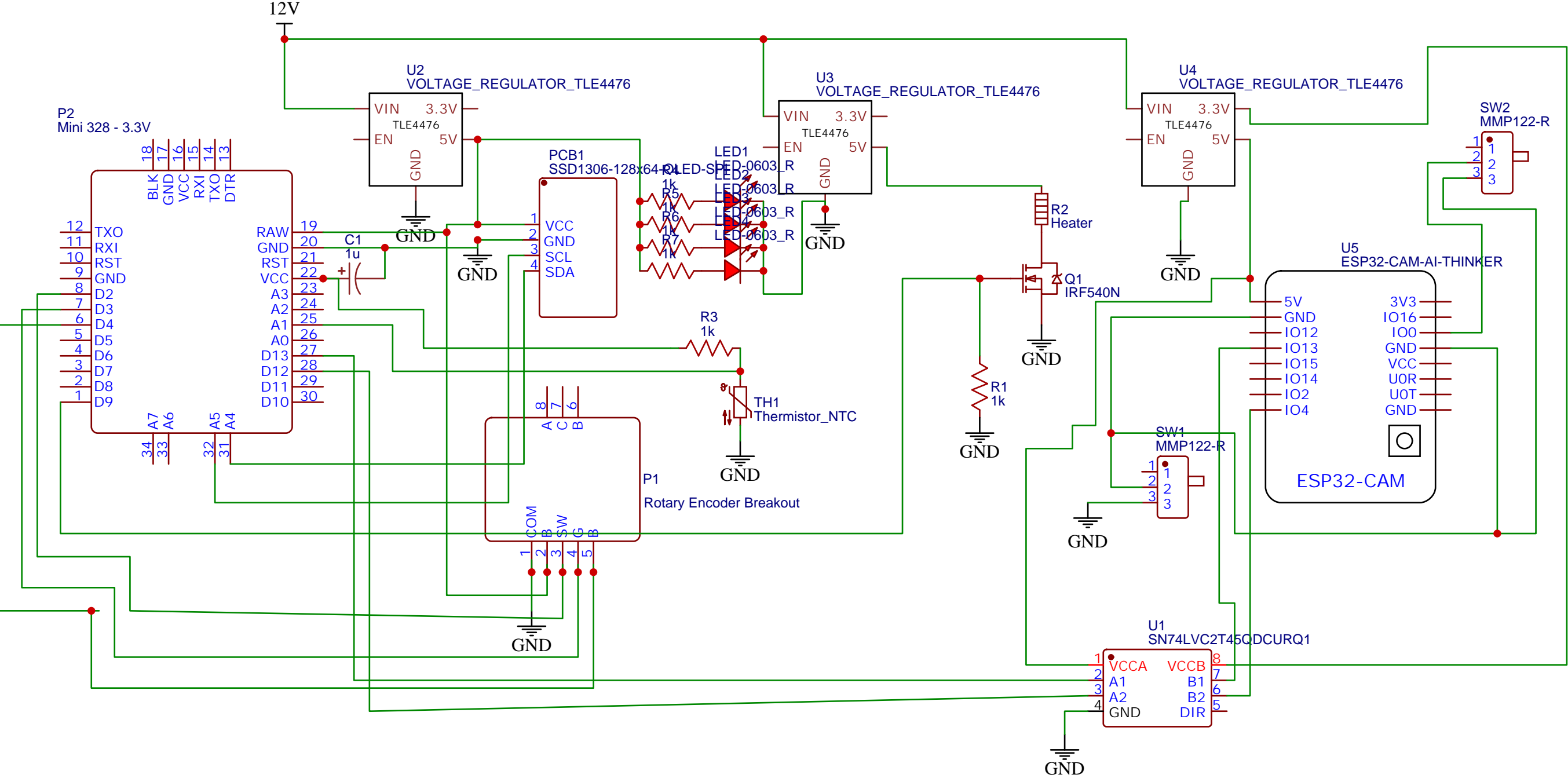

|             |                                   |            |
|-------------|-----------------------------------|------------|
| TITLE: LAMP |                                   | REV: 1.0   |
| EasyEDA     | Company:                          | Sheet: 1/1 |
|             | Date: 2022-01-28 Drawn By: saho00 |            |

Supplement: Supplementary file 2 — Supplementary Information 2. [file 41598_2023_49651_MOESM2_ESM.pdf]
