# Supplementary material for: Quantitative mRNA expression measurement at home
Source: Sci Rep. 2024 Jan 10;14:1013. doi: 10.1038/s41598-023-49651-8 (PMC10781964; doi:10.1038/s41598-023-49651-8)
Supplement: Supplementary file 4 — Supplementary Information 4. [file 41598_2023_49651_MOESM4_ESM.docx]

Supplementary Materials for

Title: Quantitative mRNA expression measurement at home

**Authors:** Sonalisa Pandey^1,2^, Sara Safa McCoy^2^, Tsering Stobdan^2^, Debashis Sahoo^1,2,3^*

**Affiliations:**

^1^Shanvi, San Diego, CA, USA.

^2^Department of Pediatrics, University of California San Diego.

^3^Department of Computer Science and Engineering, Jacob’s School of Engineering, University of California San Diego.

Correspondence to: [dsahoo@ucsd.edu](mailto:dsahoo@ucsd.edu)

**This PDF file includes:**

Materials and Methods

Supplementary Text

Figs. S1 to S6

Captions for Data S1

Captions for Data S2

Captions for Data S3

**Other Supplementary Materials for this manuscript include the following:**

Data S1

Data S2

Data S3

Materials and Methods

Tissue Samples

Human normal colon cDNA and mRNA samples were purchased from AMSBIO LLC (Colon cDNA- #C1234090; Colon mRNA- #ATR1234090-50) and Takara Bio (cDNA - #639331). Human Lung cDNA samples were purchased from Takara Bio (Lung cDNA - #639308). Human blood samples were collected as part of the University of California San Diego IRB Project#171861 (PI: Debashis Sahoo; Title: Identification of therapeutics targets of acute leukemia), and patients provided written informed consent.

Fresh tissues (Colon, Lung, Kidney, Spleen) of C57Bl/6J mice were collected in PBS for immediate processing or in RNALater and stored at -20°C. Cardiac puncture from euthanized mice was used to collect blood samples and immediately transferred to Lucigen QuickExtract solution or CPT tubes for PBMC isolation.

RT-LAMP

RT-LAMP reactions (25uL total volume) contained 1x WarmStart® Fluorescent LAMP/RT-LAMP Kit with UDG (New England Biolabs, Ipswich, MA, USA; #E1708L), a primer set composed of 1.6uM FIP/BIP internal primers, 0.4uM LF/LB loop primers and 0.2uM F3/B3 external primers, and 2uL of target samples. RT-LAMP reactions were carried out at 65°C for 45 min.

RT-PCR

A 20 μl reaction comprising 1 μl cDNA, a mixture of equal amounts of 0.5 μM forward and reverse primers, and RT-PCR master mix as recommended by the manufacturer was used. Reaction conditions were initial denaturation at 95 °C for 10 min and 40 cycles consisting of 15s at 95 °C, 1 min at 60 °C and final extension 5 s at 65 °C. The melt curve was produced from 65 to 95 with an increment of 0.5C. Runs were performed using a BioRad CFX96 RT-PCR system. PCR products were analyzed by electrophoresis on a 1.5 % agarose gel, 0.5 X TE, run at 100 V for 25 minutes, stained with GelRed, and visualized using a UV transilluminator.

Data collection and processing

A Python script was developed to analyze the SYBR green Quantification Amplification Results from BioRad CFX96. The raw fluorescence intensity was plotted as a function of time to generate the LAMP amplification curve for each chamber. The amplification curve was analyzed using 5-parameters logistic model (y = d + (a-d)/((1 + (x/c)^b)^e) where a = baseline, b = slope factor, c = inflection point, d = plateau, e = asymmetry factor(*1, 2*). Correlation tests between RT-PCR and RT-LAMP data were performed using Python seaborn package lmplots function. Fluorescence intensity was normalized using (v - min)/max formula. RT-PCR and RT-LAMP curves were plotted using Python pandas package line plot function. Welch's two-sample unpaired t-test is performed to compute the p values. Violin and swarm plots were made using Python seaborn package.

Ethics statement

Human subject research was approved by University of California San Diego IRB Project#171861 (PI: Debashis Sahoo; Title: Identification of therapeutics targets of acute leukemia) and patients provided written informed consent.

Vertebrate animal research was approved by IACUC protocol S14110 (PI: Debashis Sahoo). According to University of California San Diego Institutional Animal Care and Use Committee (IACUC) policies and guidelines, animals were bred, housed, and euthanized.

Primer design steps for CDX2 and ACTB

Another innovative aspect of LAMP is its high specificity due to the use of several primers (from four to six), which can distinguish up to eight specific locations on the DNA template, compared to only two in typical PCR. A deciding element responsible for the correct progression of the LAMP reaction is the primer design stage. Several pairs of primers must be optimized in terms of a range of factors, including concentration, location of nucleotide pairs, and distance between DNA regions. The primers must have a single-strand structure at 60–65 °C and must not create a stable double-strand structure. Using a bigger number of primers to amplify, the same sequence can increase the interactions between them. The design of LAMP primers can be carried out using online software such as PrimerExplorer (https://primerexplorer.jp/e), Premier Biosoft (<http://www.premierbiosoft.com/isothermal/lamp.html>), or NEB LAMP Primer Design Tool (https://lamp.neb.com). The pairs of primers used in LAMP are as follows: the internal primers, forward internal primer (FIP = F1c::F2) and backward internal primer (BIP = B2::B1c); the external primers, forward primer (F3) and backward primer (B3); and the optional loop primers, loop primer forward (LF) and loop primer backward (LB). To design a primer set for CDX2, sequence data from RefSeq NM_001265.6 was used (Fig. S2A). A target region with two exon boundaries (NM_001265.6:728-1311) was used to search for the LAMP primers. Two different primer sets (Primer 1: CDX2-p1, Primer 2: CDX2-p2) were chosen using the NEB LAMP Primer Design Tool (<https://lamp.neb.com>; Fig. S2A). For the human ACTB gene, two different exon boundaries were chosen (NM_001101.5: 270-511, NM_001101.5: 660-901) for the two primer sets, respectively (Primer 1: ACTB-p1, Primer 2: ACTB-p2, Fig. S2B). For the mouse Cdx2 gene, a target region that includes two exon boundaries (NM_007673.3: 713-1059) was used to search for the LAMP primers (Primer 1: Cdx2-p1, Primer 2: Cdx2-p2, Fig. S2C). The mouse Cdx2 primers differ by only the F3 sequence. For the mouse Actb gene, NM_007393.5:837-1238 target region was used to build two different primers (Primer 1: Actb-p1, Primer 2: Actb-p2, Fig. S2D). We refer to each of these primers by their names: CDX2-p1, CDX2-p2, ACTB-p1, ACTB-p2, Cdx2-p1, Cdx2-p2, Actb-p1, and Actb-p2 in this manuscript. In Addition, ten more CDX2 LAMP primer sets were designed using different segments of the CDX2 exons.

Analysis of RT-PCR and RT-LAMP data using 5PL model

Both RT-PCR and RT-LAMP require primers to maintain specificity for a particular gene. RT-PCR uses two primers (F3, B3, Fig. S2) whereas RT-LAMP uses four to six primers (F3, B3, FIP = F1c::F2, BIP = B2::B1c, LB, LF, Fig. S2) for specific target amplification. The RT-PCR quantification is performed by measuring the amount of PCR product produced at each thermocycle step of the reaction or in “real-time” by the quantity of the fluorescent signal. The point on the curve where the amount of fluorescence begins to increase rapidly, usually a few standard deviations above the baseline, is termed the cycle threshold value (Ct value). Biologically, the higher the starting copy number of the nucleic acid target is, the sooner a significant increase in fluorescence is detected and the lower the Ct value. In other words, the lower the Ct value, the higher the gene expression.

Both RT-PCR and RT-LAMP have a sigmoidal appearance when the fluorescence signal is plotted against time (in minutes; Fig. 2A). We modeled them as logistic functions with five different parameters (5PL, Fig. 2A-ii) where a = Baseline, b = slope factor, c = inflection point, d = Plateau, e = asymmetry factor(*1, 2*). These parameters are estimated using logistic regression (RT-PCR: Fig. 2A-i, RT-LAMP: Fig. 2A-iii). The inflection point can be used as a Ct value for expression quantification. Baseline, Plateau, and slope factor represent the starting value, saturation value and, the amplification rate during the exponential phase, respectively. The changes in the shape of the curve by varying a single parameter is shown in Fig. 2A-ii.

RT-LAMP amplified DNA earlier than RT-PCR and to a higher level

The LAMP primer sets ACTB-p2 (Fig. S4A-i) and CDX2-p2 (Fig. S4A-vi) were tested in four different human colon cDNA samples. The fluorescence values were plotted against time in minutes in both RT-PCR and RT-LAMP data. For both primer sets ACTB-p2 (Fig. S4A-ii) and CDX2-p2 (Fig. S4A-vii), RT-LAMP amplified DNA earlier than RT-PCR (T-tests based on the inflection points: ACTB-p2, p = 0.000132; CDX2-p2, p = 0.000917). The saturation point for RT-LAMP was higher than RT-PCR for both ACTB-p2 (Fig. S4A-iii, p = 1.1e-15) and CDX2-p2 (Fig. S4A-viii, p=1.5e-09). The slope factor for RT-LAMP was higher than RT-PCR in ACTB-p2 (Fig. S4A-iv), whereas it was the opposite in CDX2-p2 (Fig. S4A-ix). However, the asymmetry factor of RT-LAMP was consistently higher than RT-PCR in both primer sets ACTB-p2 (Fig. S4A-v) and CDX2-p2 (Fig. S4A-x). Our data agree with previous findings that LAMP increases the amount of amplified DNA even up to a billion copies over less than an hour, compared to a million copies yielded by the PCR(*3-5*).

Direct sample testing

We tested ten different processing steps of the colon tissue to check if RT-LAMP amplification was sensitive to any steps (Fig. S3B-i). We used a cotton swab to lightly scratch the two different surfaces of the colon tissue (CS1 and CS2) and immerse them in the Lucigen QuickExtract solution immediately. We put a whole chunk of the colon tissue in Lucigen QuickExtract solution (C) and stored the sample in the solution for 3 days at -20C (QE). The cotton swab samples (CS1 and CS2) were also stored in -20C for 3 days (CS1n and CS2n). A whole chunk of colon tissue was immersed in RNALater, stored at -20C for three days, and later transferred to Lucigen QuickExtract solution (RLQE1) for one minute. RLQE1 is stored at room temperature for 20 minutes and processed later (RLQE2). The colon chunk stored in RNALater was scratched using a cotton swab and processed immediately using the Lucigen QuickExtract solution (RLS1). A tiny piece of the whole colon chunk stored in Lucigen QuickExtract solution for 3 days at -20C was transferred to a fresh Lucigen QuickExtract solution for one minute (QE2).

Cotton swab on colon tissue vs whole chunk

Ten samples were subjected to RT-LAMP protocol for direct sample testing using Actb-p1 and Cdx2-p2 mouse primer set (Fig. S3B-i). Actb-p1 primer set was able to amplify mRNA in almost all samples except the colon chunk in the Lucigen QuickExtract (Fig. S3B-ii). The colon chunk

probably needs to be smaller to be processed using the limited Lucigen QuickExtract solution. We also observed that RLQE1 is amplified much earlier compared to RLQE2 (Fig. S3B-ii). This suggests that mRNAs are being rapidly degraded, ruling out inhibition of RT-LAMP activity by materials from tissue chunk in Lucigen QuickExtract solution. Accordingly, only cotton swab samples except for CS1 (opposite side from the epithelium) were amplified using the Cdx2-p2 primer set (Fig. S3B-iii). Consistent with this pattern, cotton swab samples were amplified earlier using Actb-p1 RT-LAMP (Fig. S3B-ii). Differential expression patterns between CS1 and CS2 suggest that Cdx2-p2 RT-LAMP is highly sensitive to colon epithelium. However, CS1 sample, which was stored at room temperature for a while and -20C for 3 days later called CS1n was amplified using Cdx2-p2 primer set (Fig. S3B-iii). Collectively, these data suggest that cotton swab samples performed much better compared to whole colon chunks, and mRNAs are degraded rapidly when whole colon tissue is processed using Lucigen QuickExtract solution.

3D design and printing

The 3D design was carried out using the Autodesk Tinkercad website (<https://www.tinkercad.com/>), and the designs were exported in STL format. The STL files were sliced using Ultimaker Cura 4.13.1, which generated gcode files. The gcode files were uploaded to 3D printers (Creality Ender 3 Pro 3D Printer with Removable Build Surface Plate and UL Certified Meanwell Power Supply Printing Size 8.66x8.66x9.84in, Official Creality Ender 3 V2 3D Printer Upgraded Integrated Structure Design with Silent Motherboard Mean Well Power Supply and Carborundum Glass Platform 8.66x8.66x9.84 Inch, ANYCUBIC Mega S Upgrade FDM 3D Printer with Extruder and Suspended Filament Rack 8.27''(L) x8. 27''(W) x8.07''(H) Print Size) using a microSD card. OVERTURE PLA Filament 1.75 mm PLA 3D Printer Filament, 1kg Cardboard Spool (2.2lbs), Dimensional Accuracy +/- 0.03 mm (Royal Gold) was used for printing.

PCB Design

MG Chemicals Copper Clad Board (Double Sided, 9" x 6", 1 oz Copper, 1/32" Thick, FR4) was cut to the desired shape using Dremel (8220-2/28 12-Volt Max Cordless Rotary Tool Kit with Battery) using Diamond Cutting Wheel (YEEZUGO 1/8" Titanium Coating Diamond Cutting Discs Cut-Off Wheel Blades Shank Diameter 3.00mm, Wheel diameter 1-1/2"). The 2-sided copper clad board was cleaned using steel wool. A photosensitive dry film is thermally transferred to the copper board using a GBC Thermal Laminator Machine (Fusion 7000L). PCB circuit is designed using Autodesk EAGLE software (version 9.6.2), and circuit mask is printed on transparency film using a laser printer (Github:sahoo00/LAMP). The transparency films are attached to form an aligned two-sided circuit mask using Scotch super-hold transparent tape. Copper Clad Board with photoresist film is pushed between the two-sided circuit mask and exposed to strong UV light (MelodySusie 36W Nail Polish Curing Lamp and Sliding Tray) for five minutes. The UV-exposed board is washed in soda ash (Sodium Carbonate, Na2CO3) solution and etched in Ferric Chloride for 1 hour. The etched board is washed in NaOH solution to expose the copper surface.

Supplementary Text

Extended Technical Descriptions

PCB is soldered using Aoyue 968A+ Professional SMD Digital Hot Air Rework Station with a Soldering Iron and Vacuum Pickup. The aluminum heating block is designed using six holes on a 3/8" thick 6061-T651 Aluminum Plate Custom Cut: 1 in. x 2 in. (± 1/16 in.). The holes are made using Drill Press (WEN 4214 12-Inch Variable Speed) and Step Drill Bit Set (NEIKO 10198A, 4-12 steps). PID heating controller (proportional with integral and derivative control) uses MakerHawk NTC 3950 100K Thermistor and 12V 40W 620 Ceramic Cartridge Heater. Lee filters (777-rust and 071-Tokyo Blue, [www.newlighting.com](http://www.newlighting.com), product ID 9409) were used to filter the fluorescence signal.

**Fig. S1.** Strategies for comparative analysis between RT-LAMP and RT-PCR for **mRNA expression measurement. A.** Proposed experiment to demonstrate CDX2 differential expression patterns in blood, PBMC, lung, kidney, spleen, and colon tissue. **B.** Proposed experiment to test expression patterns starting from cDNA, mRNA, and tissue quick extract samples. **C.** Proposed experiment to compare RT-PCR and RT-LAMP techniques for quantitative expression measurement using serial dilution samples.

Fig. S2. RT-LAMP Primers design steps for CDX2 and ACTB genes in human and mice.

Visualization of the LAMP primer design (F3, F2, LF, F1c, B1c, B2, B3), steps with gene model based on latest genome, and RefSeq transcript with exon annotations. Two different primers were designed for each gene. Locations of the primers were specified in the target sequence. **A.** Primers for CDX2. **B.** Primers for ACTB. **C.** Primers for Mouse Cdx2 gene. **D.** Primers for Actb gene.


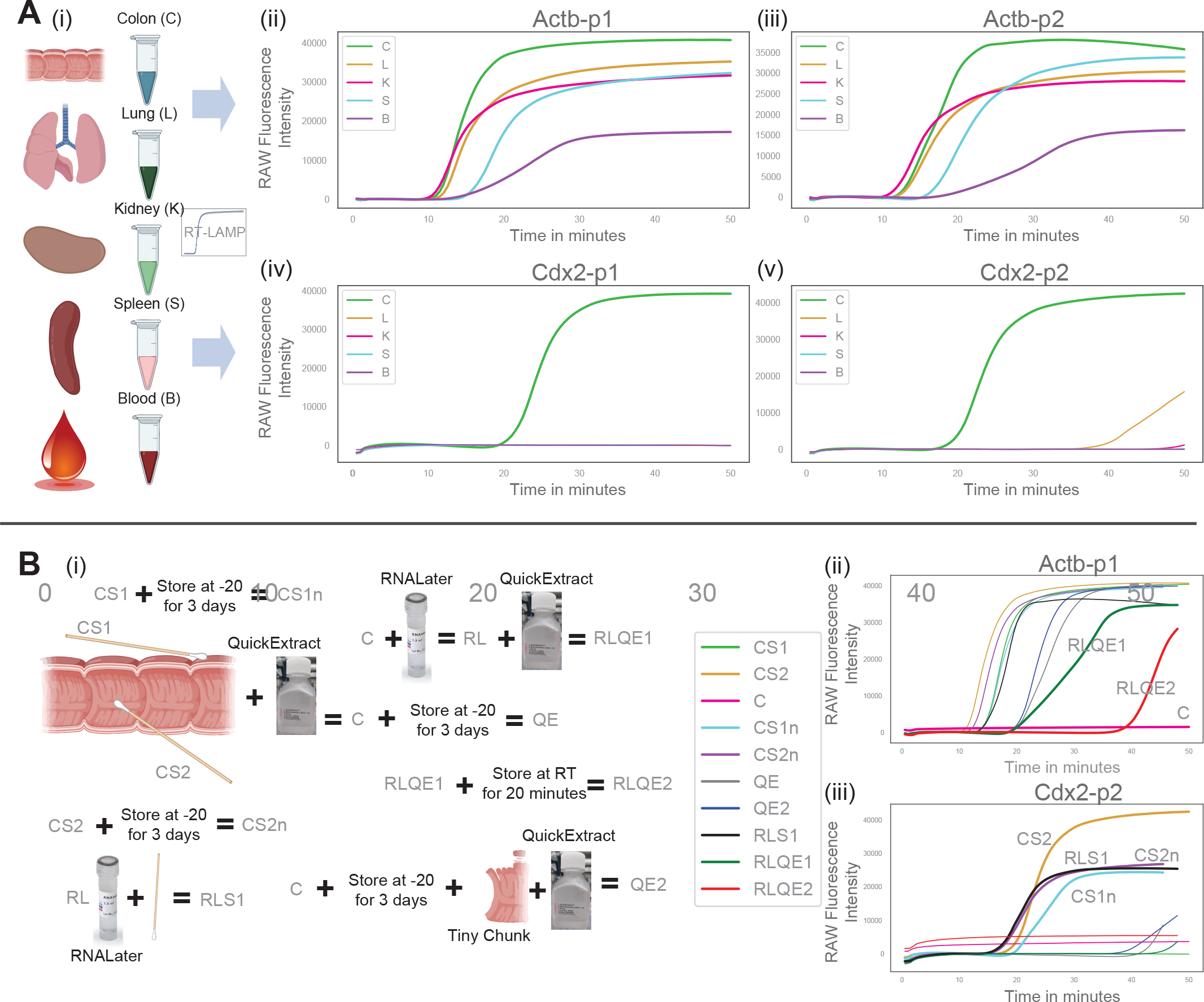


Fig. S3. Optimization of RT-LAMP based mRNA expression measurement directly from sample.

**A.** RT-LAMP experiments on direct tissue samples from five different mouse tissues (colon, lung, kidney, spleen, blood) using all four primer sets: Actb (top-left), Actb2 (top-right), Cdx2 (bottom-left), and mCdx2 (bottom-right). **B.** RT-LAMP experiment performed on colon samples prepared using 10 different protocols and two primer sets (Actb, mCdx2). CS1 is collected using a cotton swab on the opposite side of the colon epithelium (-ve control for Cdx2 expression) and processed using Lucigen QuickExtract solution. CS2 is collected using a cotton swab directly on the colon epithelium. C is a big chunk of whole colon tissue dropped in the Lucigen QuickExtract solution. CS1n and CS2n are the same CS1 and CS2 samples stored at -20C for 3 days, respectively, and re-used for the RT-LAMP experiment. QE is the same as sample C stored at -20C for 3 days and re-used for the RT-LAMP experiment. A tiny chunk of colon tissue is transferred to a new QuickExtract solution from sample C stored at -20C for 3 days for the QE2 sample. A whole chunk of colon tissue was stored in RNALater at -20C for 3 days and re-used to make RLS1, RLQE1, and RLQE2 samples. RLS1 is collected using a cotton swab on the whole colon chunk from the RNALater solution. A small chunk of colon tissue from the RNALater solution is dropped into a fresh QuickExtract solution to make an RLQE1 sample and processed quickly after 1 minute. RLQE2 sample is the same as the RLQE1 sample processed after 20 minutes.


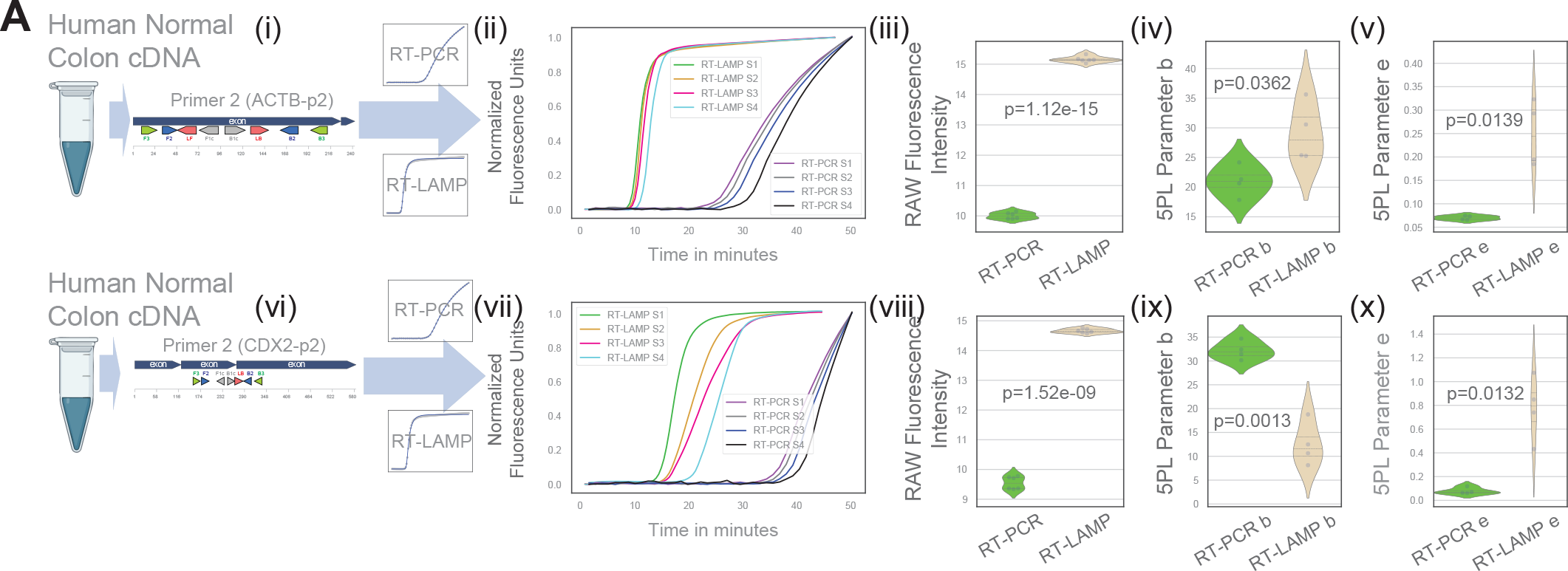


**Fig. S4.** **Rapid mRNA expression measurement using RT-LAMP and comparison with RT-PCR.** **A.** Human normal colon cDNA samples were used in RT-PCR and RT-LAMP experiments. ACTB-p2 and CDX2-p2 LAMP primers set were used for the RT-LAMP experiment. F3 and B3 primers (ACTB-p2 and CDX2-p2) were used to perform the RT-PCR experiment. RT-PCR data is directly compared to RT-LAMP data on the same plot with the same time scale and normalized fluorescence intensity ranging from 0 to 1. Using violin plots RAW fluorescence intensities, 5PL b, and e values are compared between RT-PCR and RT-LAMP.


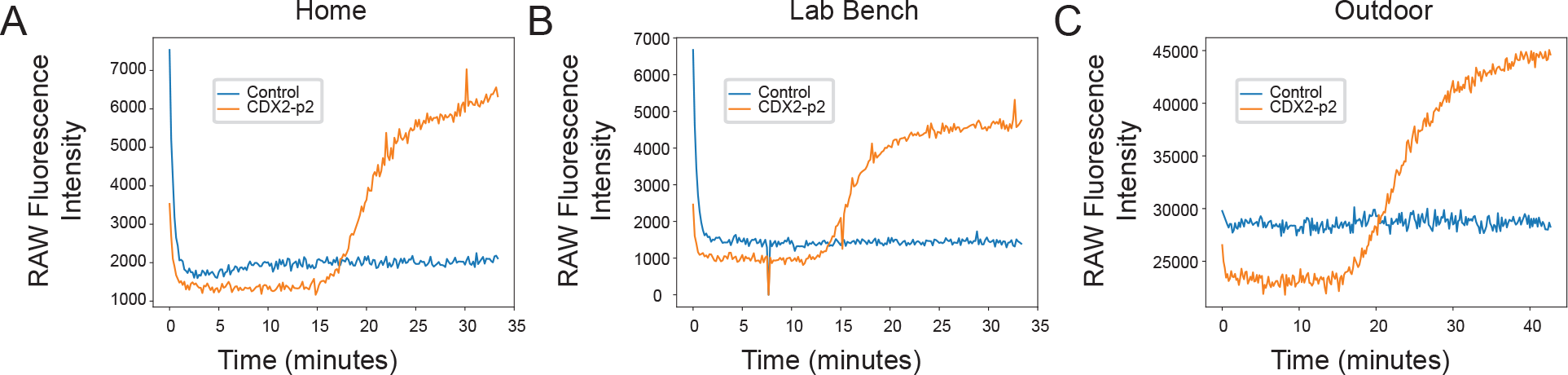


Fig. S5. CDX2 expression measurement at home, laboratory bench top, and outdoor using a hardware device

(**A-C**). RT-LAMP primer CDX2-p2 amplifies colon mRNA samples at home (A), laboratory bench top (B), and outdoor setting (C).


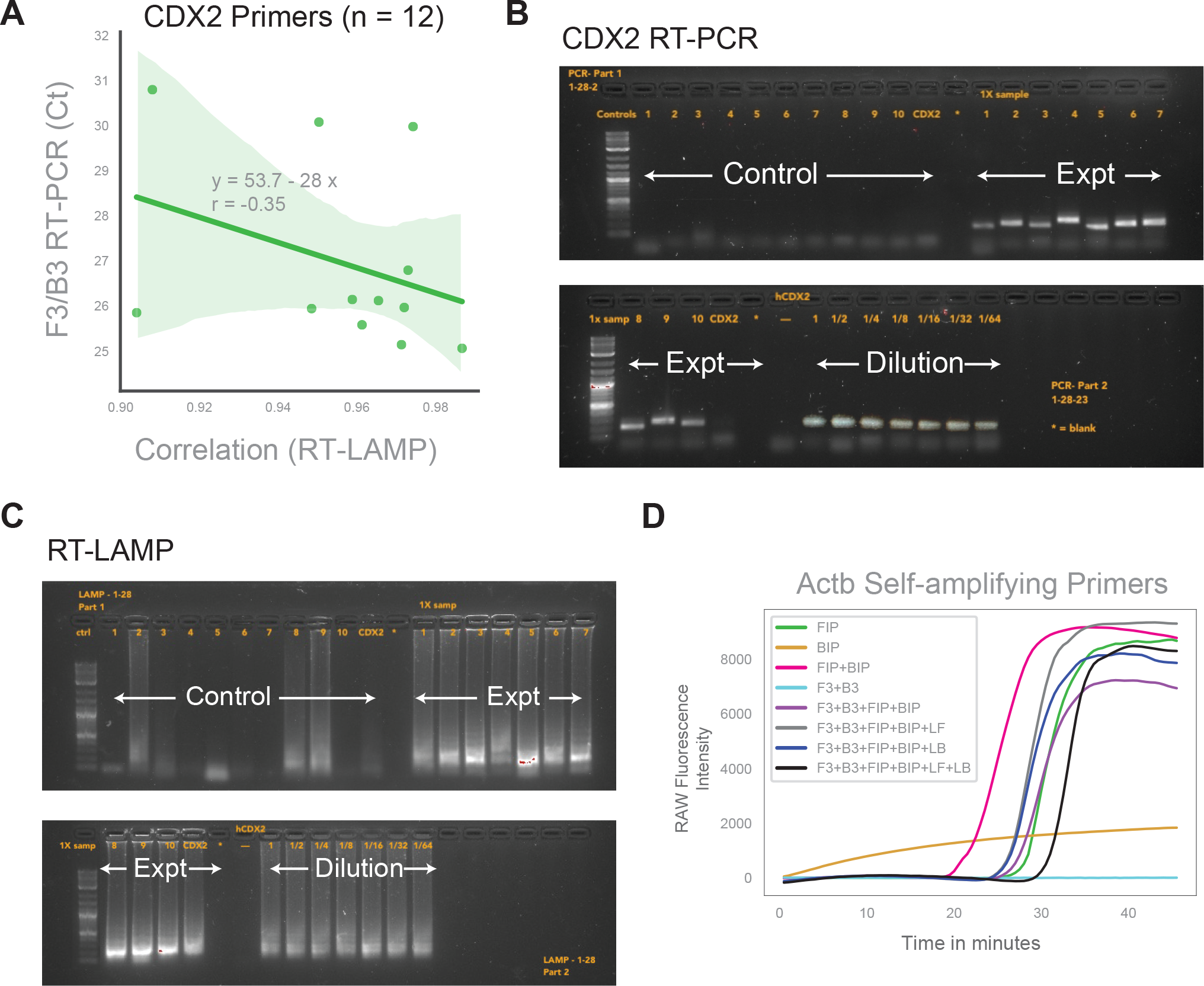


Fig. S6. LAMP Primer optimizations and self-amplification.

**A.** Twelve different RT-LAMP primers (Supplementary Data 3) are used on six different serial dilutions of colon mRNA samples. Correlation coefficient between the inflection points and dilutions is plotted on the x-axis. Y-axis represents the RT-PCR inflection point (Ct) of the F3/B3 parts of the RT-LAMP primers. Each point in the plot represents an RT-LAMP primer. **B.** Full-length GEL run from the RT-PCR experiment (Control, Expt for 11 CDX2 primers, and serial dilution of CDX2-p2 primer). The dot represents no sample. **C.** Full-length GEL run from the RT-LAMP experiment (Control, Expt for 11 CDX2 primers, and serial dilution of CDX2-p2 primer). The dot represents no sample. **D.** Detailed analysis of a self-amplifying Mouse Actb LAMP primer specific for DNA. The RT-LAMP assay is run without any mouse DNA sample in the experiment. FIP and BIP alone can self-amplify for this primer set, whereas F3+B3 cannot.

Data S1. (separate file)

RT-PCR and RT-LAMP primer sequences used in this manuscript.

Data S2. (separate file)

Circuit Schematic diagram for the LAMP hardware device.

Data S3. (separate file)

Sequence details of Twelve different CDX2 RT-LAMP primers and one mouse Actb DNA specific primer to demonstrate the self-amplification problem.

**REFERENCES**

1. W. N. Cumberland *et al.*, Nonlinear Calibration Model Choice between the Four and Five-Parameter Logistic Models. *J Biopharm Stat* **25**, 972-983 (2015).

2. A. N. Spiess, C. Feig, C. Ritz, Highly accurate sigmoidal fitting of real-time PCR data by introducing a parameter for asymmetry. *BMC Bioinformatics* **9**, 221 (2008).

3. T. Notomi *et al.*, Loop-mediated isothermal amplification of DNA. *Nucleic Acids Res* **28**, E63 (2000).

4. M. Parida, S. Sannarangaiah, P. K. Dash, P. V. Rao, K. Morita, Loop mediated isothermal amplification (LAMP): a new generation of innovative gene amplification technique; perspectives in clinical diagnosis of infectious diseases. *Rev Med Virol* **18**, 407-421 (2008).

5. M. Soroka, B. Wasowicz, A. Rymaszewska, Loop-Mediated Isothermal Amplification (LAMP): The Better Sibling of PCR? *Cells* **10**, (2021).
